# Mesenchymal stem cell-derived extracellular vesicles in diabetes: immunomodulation and therapeutic prospects

**DOI:** 10.3389/fimmu.2026.1918124

**Published:** 2026-10-19

**Authors:** Lingli Ma, Ning Hou, Xuejiao Zhang, Xiaoyu Zhao, Qing Liu, Yan Zhang, Jiajing Dong, Zimeng Li, Qing Wang

**Affiliations:** 1 Department of endocrinology and metabolism, China-Japan Union Hospital of Jilin University, Changchun, China; 2 Department of Cardiac Superficial Musculoskeletal Ultrasound, Songyuan Central Hospital, Songyuan, China

**Keywords:** diabetes, diabetic complications, immune regulation, MSC-derived extracellular vesicles, therapeutic mechanisms

## Abstract

Diabetes mellitus is characterized by chronic hyperglycemia, immune dysregulation, and progressive multi-organ injury. Mesenchymal stem cell-derived extracellular vesicles(MSC-EVs) have emerged as cell-free mediators capable of transferring bioactive cargo and modulating immune, metabolic, and regenerative responses. This narrative review summarizes current evidence on the roles of MSC-EVs in diabetes, focusing on immune-cell regulation, pancreatic β-cell protection, metabolic homeostasis, and major complications, while also considering EV isolation, engineering, delivery, and clinical translation. Preclinical studies indicate that MSC-EVs can modulate T-cell, macrophage, dendritic-cell, B-cell, natural killer-cell, innate lymphoid-cell, and complement-associated responses; reduce inflammatory and oxidative injury; protect β cells from apoptosis; improve insulin signaling; and promote tissue repair in diabetic wounds, kidney disease, retinopathy, neuropathy, cardiovascular injury, bone metabolic disorders, and erectile dysfunction. These effects are mediated by preparation-specific cargo and signaling pathways, including miRNAs, proteins, programmed death-ligand 1(PD-L1)/PD-1 signaling, Phosphoinositide 3-kinase/Protein Kinase B(PI3K/Akt)-related pathways, and phosphatase and tensin homolog(PTEN)/AKT signaling. The review also distinguishes direct diabetes-specific evidence from mechanistic background derived from non-diabetic models. However, the evidence remains heterogeneous across MSC sources, EV preparations, experimental models, doses, and delivery strategies, and current clinical evidence is still limited. Registered investigations should therefore be distinguished from studies reporting clinical outcomes, and product-specific efficacy should not be inferred from trial registration alone. Overall, MSC-EVs represent a promising but not yet established therapeutic platform for diabetes. Future translation will require standardized terminology and characterization, scalable manufacturing, reproducible potency assessment, optimized targeting and dosing, long-term safety evaluation, and well-designed product-specific clinical trials.

## Graphical Abstract

**Figure:**
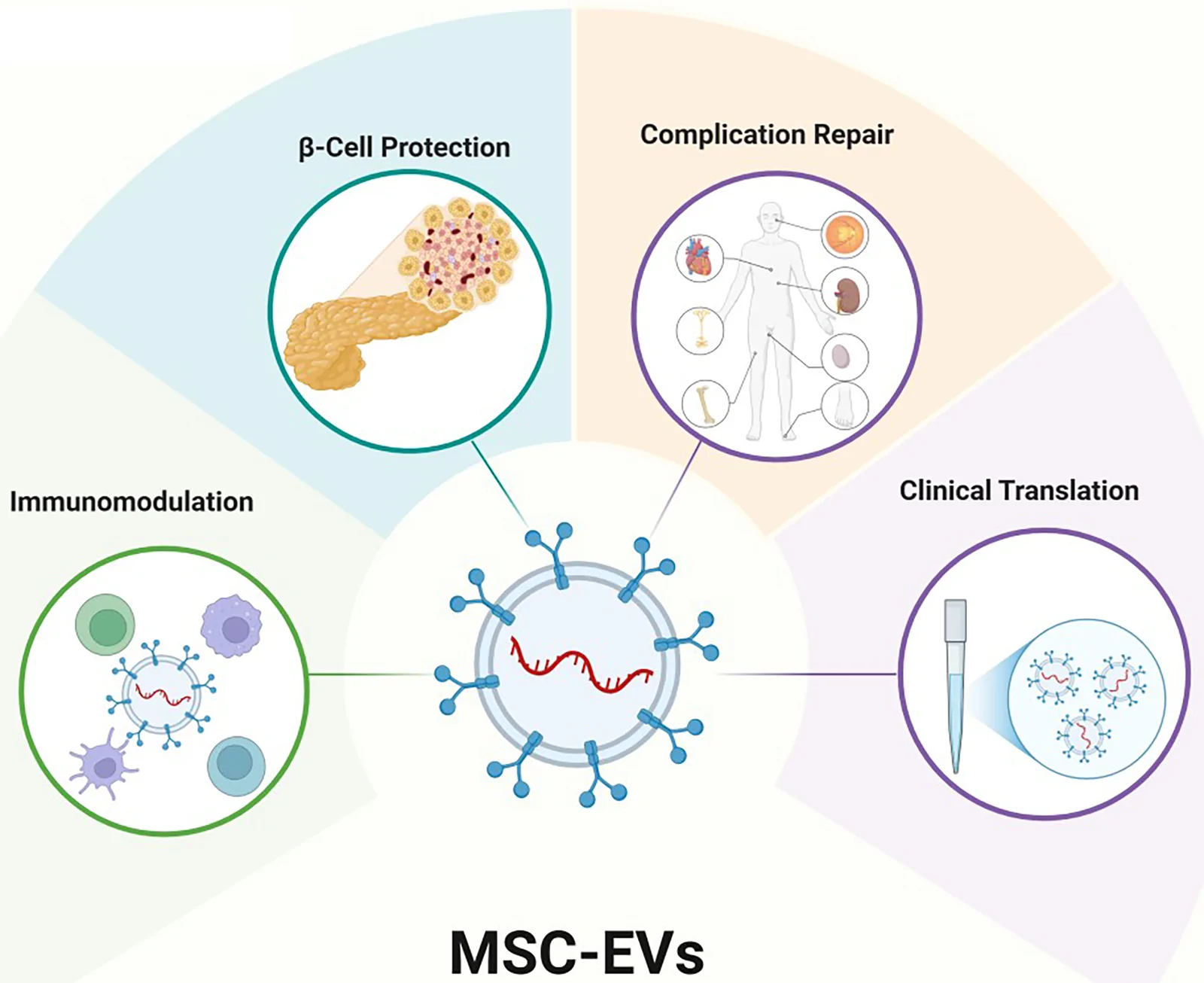

## Introduction

Diabetes mellitus is a complex metabolic disorder characterized by chronic hyperglycemia and immune dysregulation (1, 2). Its pathogenesis involves abnormal immune responses and chronic inflammation, which collectively contribute to pancreatic β-cell dysfunction and the development of multi-organ complications. The global prevalence of diabetes has reached epidemic proportions, with diabetic retinopathy(DR), nephropathy, and neuropathy emerging as major causes of morbidity and premature mortality (3, 4). Current therapeutic strategies, including insulin replacement and oral hypoglycemic agents, primarily focus on glycemic control but show limited efficacy in ameliorating immune abnormalities and promoting tissue repair (5). This unmet clinical need has spurred intensive research into novel interventions, particularly stem cell-based therapies, which offer regenerative potential through the paracrine secretion of bioactive factors with immunomodulatory, anti-inflammatory, and pro-healing properties. However, challenges such as poor cell survival, immune rejection, and tumorigenic risks associated with stem cell transplantation have necessitated the exploration of cell-free alternatives, with mesenchymal stem cell-derived extracellular vesicles(MSC-EVs) emerging as a promising candidate (6).

MSC-EVs mediate intercellular communication through the transfer of bioactive cargo (7, 8). Unlike their parent cells, MSC-EVs exhibit low immunogenicity, high stability, and scalable production capacity, while retaining the therapeutic benefits of MSCs in modulating immune responses, enhancing angiogenesis, and reducing oxidative stress. Preclinical studies have demonstrated that MSC-EVs effectively improve β-cell function, alleviate insulin resistance, and promote wound healing in diabetic models through mechanisms involving the activation of the Phosphoinositide 3-kinase/Protein Kinase B(PI3K/Akt) pathway, suppression of NOD-like receptor pyrin domain-containing protein 3(NLRP3) inflammasomes, and induction of M2 macrophage polarization (9, 10). Notably, MSC-EVs derived from different tissues (e.g., bone marrow, adipose, umbilical cord) display distinct molecular profiles and therapeutic potentials, highlighting their heterogeneity and context-dependent efficacy (**Table 1**).

**Table 1:** Comparison of mesenchymal stem cells and their extracellular vesicles in the treatment of diabetes.

Comparison dimension	Mesenchymal stem cells (MSCs)	MSC-derived extracellular vesicles (MSC-EVs)
Cell Source	Derived from bone marrow, adipose tissue, umbilical cord, and other tissues. Functional properties vary with tissue source and culture conditions.	Released by MSCs from the corresponding tissue source and containing selected donor-cell-derived components. Their composition and biological activity depend on the donor cells and culture conditions.
Mechanism	1.Human bone marrow-, adipose tissue-, and umbilical cord Wharton’s jelly-derived MSCs have been induced into insulin-producing cells under defined *in vitro* conditions (11–13); 2. Paracrine mediators include TGF-β1 and VEGF, with secretion profiles varying among bone marrow-, adipose tissue-, and Wharton’s jelly-derived MSCs (14, 15); 3. Interactions with immune cells can suppress T-cell proliferation and modulate macrophage phenotype (16, 17).	1. EV cargo includes miRNAs, proteins, and lipids. Examples include miR-146a in engineered bone marrow MSC-EVs, miR-26a-5p in adipose MSC-EVs, and PD-L1 or SIRT1 in selected engineered preparations, 2. TLR4/NF-κB suppression and macrophage phenotype modulation have been reported in specific experimental models; 3. Akt-associated insulin signaling and Wnt/β-catenin-associated bone repair have been reported in distinct diabetic models.
Immunogenicity	Not intrinsically immune privileged; allogeneic immune rejection can occur.	MHC class I has been detected in some preparations, and MSC-EVs can activate innate immune signaling. Their cell-free nature does not establish absence of immunogenicity; immune responses require preparation-specific assessment.
Safety	An updated meta-analysis of intravascular MSC trials found increased fever but no detected increase in malignancy. Safety conclusions remain limited to the products, populations, and follow-up evaluated	Cell-free and unable to replicate independently, but not inherently risk-free. Long-term safety and potential adverse effects require preparation-specific evaluation.
Tissue Targeting	Intravenous administration can result in pulmonary first-pass retention, limiting delivery to downstream tissues.	Biodistribution depends on cell source and administration route. Engineering or carrier-assisted strategies can improve targeted delivery in specific experimental settings.
Production and Storage	Requires controlled cell expansion and validated cryopreservation procedures, with assessment of post-thaw viability and functional activity.	Scalable production has been demonstrated for selected MSC-EV products. Separation quality and storage stability depend on the methods, formulation, and storage conditions.
Durability of Efficacy	Cell persistence can be brief after intravenous administration in animal models; persistence alone should not be equated with the duration of therapeutic benefit.	Repeated dosing has been used in diabetic neuropathy studies, and hydrogel delivery can sustain local EV release. These findings do not establish a universal duration of therapeutic benefit.
Effect on Diabetic Complications	Tissue-repair effects have been investigated in diabetic wound models (18). Bone marrow MSC therapy has also been evaluated in patients with diabetic critical limb ischemia and foot ulcers.	Reduced renal fibrosis and improved neuropathy outcomes have been reported in diabetic animal models using specific MSC-EV preparations. These findings are preclinical and do not establish efficacy across all diabetic complications.
Clinical Translation Potential	Evaluated in clinical studies of T1D and diabetic ischemic foot disease. Findings remain specific to the cell product, patient population, and trial design.	A cell-free therapeutic approach supported by preclinical studies. Clinical translation requires standardized manufacturing, quality control, and product-specific evaluation of safety and efficacy.

BMSCs stands for Bone Marrow Mesenchymal Stem Cells; ADSCs stands for Adipose-Derived Stem Cells; hUCMSC-EVs stands for Human Umbilical Cord Mesenchymal Stem Cell-Derived Extracellular Vesicles; miR stands for microRNA; PD-L1 stands for Programmed Death-Ligand 1; SIRT1 stands for Sirtuin 1; TLR4 stands for Toll-Like Receptor 4; NF-κB stands for Nuclear Factor-Kappa B; PI3K stands for Phosphatidylinositol 3-Kinase; Akt stands for Protein Kinase B; TGF-β stands for Transforming Growth Factor-Beta; VEGF stands for Vascular Endothelial Growth Factor; MHC stands for Major Histocompatibility Complex. MSC-EVs is used here as an umbrella term and does not imply that all preparations are exosomes or have the same size distribution. This table summarizes findings from different studies and does not establish that MSCs or MSC-EVs are uniformly superior in efficacy, safety, targeting, or durability. General methodological studies are used for context rather than as direct evidence of efficacy in diabetes.

Human bone marrow and adipose tissue MSCs yielded insulin-producing cells under the same induction protocol, with no significant difference in glucose-stimulated insulin or C-peptide release in a small comparison involving two donors per source (12). In a separate *in vitro* study, differentiated Wharton’s jelly stromal cells showed higher C-peptide positivity and insulin secretion than differentiated bone marrow MSCs (13). These findings identify candidate sources for induction but do not establish a universally superior source for clinical β-cell replacement.

For paracrine activity, transforming growth factor-β1(TGF-β1) was detected in bone marrow, adipose tissue, and Wharton’s jelly MSC cultures, without significant differences among these sources in one comparison (15). Another study found higher vascular endothelial growth factor A(VEGF-A) secretion from bone marrow MSCs but higher hepatocyte growth factor secretion from Wharton’s jelly MSCs under the tested conditions (14). Source selection should therefore consider the intended functional endpoint and culture conditions rather than assume a single optimal source. These donor-cell findings should not be interpreted as demonstrating corresponding differences in EV cargo or therapeutic potency.

The additional mechanisms summarized in **Table 1** concern distinct EV preparations and therapeutic goals. Cytokine-primed MSC-EVs loaded with hexyl 5-aminolevulinate hydrochloride(HAL) and expressing high levels of programmed death-ligand 1(PD-L1) reduced CD4+ T-cell activation through PD-L1/PD-1 signaling and promoted M2-associated macrophage responses in a type 1 diabetes(T1D) mouse model (19). EVs from sirtuin 1(SIRT1)-overexpressing bone marrow MSCs supported mitochondrial function in diabetic neuropathy (20). Human umbilical cord MSC-EVs improved Akt-associated insulin signaling and reduced β-cell apoptosis in rats with experimentally induced type 2 diabetes(T2D) (21). In diabetic osteoporosis(DOP), magnetic nanoparticle-assisted delivery of bone marrow MSC-EVs linked miR-150-5p-mediated matrix metalloproteinase 14(MMP14) inhibition to Wingless-type MMTV integration site family/β-catenin signaling(Wnt/β-catenin) activation and osteogenesis (22). These preparation-specific findings support investigation of β-cell preservation, improved insulin responsiveness, and complication repair, but do not establish a cure for diabetes.

The therapeutic advantages of MSC-EVs extend beyond their biological functions to address critical translational challenges. MSC-EV products are undergoing clinical evaluation, and their therapeutic potential should be interpreted in relation to trial-specific evidence of safety and efficacy rather than registration alone. Despite this progress, hurdles remain, including standardization of isolation protocols, batch-to-batch variability, and long-term safety evaluation (23). This review aims to systematically summarize the latest research advancements regarding the immunomodulatory roles of stem cell-derived EVs in diabetes, focusing on their mechanisms of action, therapeutic potential, and the challenges that lie ahead in their clinical application. By elucidating the multifaceted roles of these EVs in regulating immune responses and promoting tissue regeneration, we hope to provide insights into their potential as a transformative approach to diabetes treatment and management.

## Literature search and selection

Literature searches were conducted in PubMed, Web of Science Core Collection, Scopus, and Embase for publications dated on or before 22 September 2026, without a lower publication-date limit. The core search combined the following title and abstract terms, with syntax adapted to each database: (“mesenchymal stem cell*” OR “mesenchymal stromal cell*” OR “adipose-derived stem cell*” OR “bone marrow stromal cell*”) AND (“extracellular vesicle*” OR exosom* OR microvesicle*) AND (diabet* OR hyperglyc* OR “insulin resistance”). Full-length original studies investigating MSC-EVs in diabetes or its complications, including cellular, animal, and human studies, were considered for the therapeutic evidence synthesis. Relevant publications on EV biology, immunomodulation, isolation, engineering, and delivery, including foundational studies, reviews, and consensus recommendations, were considered for background and methodological discussion. Studies involving other EV sources were used for contextual or comparative purposes and were distinguished from direct evidence of MSC-EV therapeutic effects. Duplicate records, conference abstracts without full reports, and publications unrelated to the scope of the review were excluded. Literature selection was guided by relevance to the review questions, and the findings were synthesized narratively.

## Pathophysiological changes and immune-related pathogenesis of diabetes

Diabetes mellitus is a complex metabolic disorder characterized by chronic hyperglycemia. Its pathophysiological changes are multifaceted and closely related to immune-associated pathogenesis. In T1D, it is primarily an autoimmune disease. Genetic susceptibility to T1D is strongly associated with variation in the human leukocyte antigen(HLA) region, particularly the HLA-DR/DQ loci involved in antigen presentation (24). Prolonged enterovirus B infection has been associated with islet autoimmunity in genetically susceptible children, supporting a possible environmental contribution rather than a universal causal trigger of T1D (25). Once activated, T lymphocytes, along with B lymphocytes and macrophages, recognize specific β-cell antigens in the pancreas as foreign (26). This leads to the destruction of insulin-producing β-cells, resulting in severely impaired insulin secretion and subsequent hyperglycemia. The persistent immune attack not only damages β-cells directly but also disrupts the normal pancreatic microenvironment, further exacerbating the loss of β-cell function (27).

The pathogenesis of T2D is quite complex. Obesity, physical inactivity, aging, and genetic susceptibility are established contributors to T2D risk, although their relative contributions vary across individuals and populations (28, 29). Initially, insulin resistance develops, causing peripheral tissues such as muscle, adipose tissue, and the liver to become less sensitive to insulin. This triggers the pancreas to produce more insulin to keep blood glucose levels normal, resulting in hyperinsulinemia (28). Over time, the pancreatic β-cells become overworked and gradually lose their function, a condition referred to as β-cell exhaustion (30). Chronic, low-grade inflammation is also a significant characteristic of T2D (31). Elevated levels of inflammatory cytokines, including tumor necrosis factor-α(TNF-α), interleukin-6(IL-6), and C-reactive protein(CRP), are frequently observed. These cytokines can disrupt insulin signaling pathways, thereby promoting insulin resistance (32, 33). Macrophages in adipose tissue, particularly in obese individuals, are activated and tend to polarize towards the pro-inflammatory M1 phenotype. They secrete large quantities of pro-inflammatory mediators, which contribute not only to local adipose tissue inflammation but also have systemic effects on insulin sensitivity and glucose metabolism (34, 35). Furthermore, the production of advanced glycation end products(AGEs) due to chronic hyperglycemia can activate the immune system by binding to their receptors for AGEs, initiating a cascade of inflammatory responses and oxidative stress. These processes, in turn, accelerate the progression of T2D and its complications (36).

## Immunological distinctions between T1D and T2D and implications for MSC-EV therapy

T1D and T2D differ in the context in which immune dysregulation develops. T1D involves β-cell-directed autoimmunity, with a particularly strong contribution from HLA-DR/DQ variation and possible environmental influences such as enterovirus infection (24, 25). By contrast, T2D has a heterogeneous genetic architecture involving β-cell function, adiposity, and insulin-resistance pathways (29). In obesity-associated T2D, metabolic tissue stress provides an important context for inflammatory activation, rather than the HLA-associated loss of tolerance to islet antigens that characterizes T1D. These distinctions concern predominant disease mechanisms and should not be interpreted as a single initiating event shared by all individuals with either condition.

Islet-antigen presentation and autoreactive CD4+ and CD8+ T-cell responses are central to T1D, with antigen-presenting cells participating in the initiation and maintenance of these responses (37). Human-islet experiments have shown that IL-17 potentiates β-cell apoptosis induced by interferon-γ(IFN-γ) combined with IL-1β or TNF-α (38). In obesity-associated T2D, macrophage accumulation and inflammasome activation connect metabolic stress to chronic inflammation and impaired insulin action. Adipose macrophages contribute TNF-α and IL-6, while NLRP3 inflammasome activity links obesity-associated danger signals to inflammatory cytokine production and insulin resistance (39, 40). Thus, the contrast is not simply adaptive immunity in T1D versus innate immunity in T2D; it lies in the predominant cellular interactions, tissue targets, and organization of the inflammatory response.

These differences imply distinct therapeutic priorities for MSC-EVs rather than entirely separate mechanisms. In T1D, the expected priorities are to restrain autoreactive T-cell responses, support regulatory antigen presentation, and preserve residual β cells. MSC-EV-conditioned dendritic cells(DCs) from patients with newly diagnosed T1D reduced inflammatory T-cell responses to glutamic acid decarboxylase 65(GAD65) ex vivo (41). In T2D, the anticipated emphasis is on reducing metabolic inflammation while improving peripheral insulin responsiveness and protecting β-cell function. Human umbilical cord MSC-EVs improved insulin signaling and reduced β-cell apoptosis in rats rendered diabetic by a high-fat diet and streptozotocin (21). These findings support disease-informed mechanistic hypotheses, but do not establish mutually exclusive actions of MSC-EVs or demonstrate that improved glycemia alone represents restoration of immune tolerance.

## Biological characteristics and separation techniques of MSC-EVs

### Terminology and evidence interpretation under MISEV2023

Following the Minimal Information for Studies of Extracellular Vesicles 2023 (MISEV2023) recommendations, we use MSC-EVs as an umbrella term without implying a specific biogenesis pathway. The designation mesenchymal stem cell-derived small extracellular vesicles(MSC-sEVs) is used when reported size measurements support a small-EV population, commonly described operationally as less than 200 nm in diameter. When size information is insufficient, the broader term MSC-EVs is retained. The term “exosome” is reserved for vesicles with demonstrated endosomal origin; particle size or detection of common EV-associated proteins alone does not establish this origin (23).

Relevant methodological considerations include the MSC source and culture conditions, separation procedures, particle size and morphology, EV-associated proteins, non-vesicular co-isolates, and functional controls that distinguish EV-associated activity from the effects of other preparation components (42). These recommendations should be applied according to the experimental system and research question, rather than as a uniform checklist requiring identical assays across studies. Criteria for identifying donor MSCs should be distinguished from recommendations for characterizing their EVs and establishing EV-associated biological activity (42–44).

Given its focus on immunomodulatory mechanisms and therapeutic evidence, this narrative review uses MISEV2023 to guide terminology and evidence interpretation rather than to provide an exhaustive item-by-item compliance assessment of every cited study. Such an assessment depends on the availability and completeness of methodological information in the original articles and **Supplementary Materials**. Where essential characterization or functional-control data are not reported, adherence to the relevant recommendations and attribution of observed effects to a defined EV population remain uncertain. Missing information does not establish that a procedure was not performed, but it limits the strength of mechanistic conclusions and comparisons across studies.

### Structural composition and functions of MSC-EVs

MSC-EVs are heterogeneous, lipid bilayer-delimited particles released by MSCs. Their membranes enclose luminal cargo and present molecules that can participate in interactions with recipient cells (23, 45). MSC-EV preparations contain proteins, nucleic acids, and lipids, with composition varying according to source and preparation conditions (45, 46). Tetraspanins such as Cluster of differentiation 9(CD9), CD63, and CD81 are commonly examined EV-associated proteins; their detection does not, by itself, demonstrate an endosomal origin (47). Furthermore, exosomes contain various functional proteins, including heat shock proteins(HSPs) that protect proteins from denaturation and assist in protein folding, thereby maintaining cellular homeostasis (48). MSC-EV preparations can also contain metabolic enzymes, signaling proteins, and cytokine-associated cargo, although their abundance and functional relevance vary with the cell source and preparation conditions (45, 49).

Nucleic acids are a key component of stem cell-derived exosomes. MicroRNAs(miRNAs), a class of small non-coding RNAs, are present in abundance (50, 51). MiRNAs can regulate gene expression at the post-transcriptional level by binding to complementary sequences on target messenger RNAs(mRNAs), leading to mRNA degradation or translational repression. MSC-EV-associated miRNAs have been implicated in distinct cellular responses. EVs from human synovial MSCs overexpressing miR-126-3p enhanced dermal microvascular endothelial-cell proliferation *in vitro (*52). MiR-140-3p-enriched EVs from rat bone marrow MSCs promoted osteogenic differentiation of bone marrow MSCs from diabetic rats, with mechanistic evidence identifying plexin B1(Plxnb1) as a miR-140-3p target (53). EVs from mouse adipose-derived MSCs transferred miR-26a-5p to high-glucose-exposed mouse podocytes and reduced apoptosis by targeting Toll-like receptor 4(TLR4) (54). In addition to miRNAs, MSC-EVs can carry long non-coding RNAs (lncRNAs). EVs from human hair follicle MSCs overexpressing H19 reduced NLRP3-associated pyroptosis in high-glucose-exposed HaCaT keratinocytes and promoted wound healing in diabetic mice (55). EVs from human bone marrow MSCs engineered to overexpress HOX transcript antisense RNA(HOTAIR) enhanced angiogenesis and wound healing in db/db mice, while EV-treated endothelial cells showed increased HOTAIR and VEGF expression (56).

Lipids in exosomes are not only the building blocks of the bilayer membrane but also have important biological functions. Phospholipids, cholesterol, and sphingolipids are the main lipid components. The lipid composition of exosomes is distinct from that of the plasma membrane of the parent cells, which may endow exosomes with unique physical and chemical properties. For example, the specific lipid composition may affect the fluidity and stability of the exosomal membrane, as well as its interaction with target cells (57).

Functionally, stem cell-derived exosomes play a pivotal role in cell-to-cell communication and tissue homeostasis. They can be taken up by recipient cells through various mechanisms, including endocytosis, membrane fusion, or receptor-mediated uptake (49). Following uptake, EV-associated cargo can influence intracellular signaling and recipient-cell phenotypes, although the route and efficiency of cargo delivery vary across experimental systems (58). In preclinical models of diabetes and its complications, MSC-EVs exhibit immunomodulatory functions. They can regulate the activation and function of immune cells. For instance, MSC-EV treatment has been associated with reduced pro-inflammatory macrophage markers and cytokine production, together with increased M2-associated markers (59, 60). These findings support a potential role in alleviating diabetes-associated inflammation.

Stem cell-derived exosomes also play a crucial role in promoting tissue repair and regeneration. In damaged tissues, exosomes can deliver growth factors, such as VEGF, fibroblast growth factor(FGF), and insulin-like growth factor(IGF), to recipient cells. These growth factors can stimulate cell proliferation, migration, and differentiation, thereby promoting the repair of damaged tissues (61). For example, in diabetic wound healing, exosomes can enhance the proliferation and migration of keratinocytes and fibroblasts, accelerate angiogenesis, and ultimately promote the closure of wound tissues (62). Moreover, exosomes can regulate cell metabolism. They can transfer metabolic enzymes and regulatory factors to recipient cells, influencing intracellular metabolic pathways. In cases of diabetes, where metabolic disorders are prominent, exosomes may help to correct abnormal metabolism by modulating key metabolic processes, such as glucose uptake, glycolysis, and lipid metabolism (63).

Early work in a non-diabetic acute kidney injury model showed that human bone marrow MSC-derived microvesicles promoted renal recovery and that RNase treatment abolished their protective activity, supporting an RNA-dependent contribution to paracrine tissue repair (64).

### Secretory pathways and regulatory mechanisms of EVs

EVs arise through different pathways, including outward budding from the plasma membrane and formation of intraluminal vesicles within endosomal multivesicular bodies (MVBs). The latter vesicles are released as exosomes when MVBs fuse with the plasma membranet (65, 66). For instance, in high-glucose conditions, EV secretion is significantly reduced in proximal tubule cells of diabetic mice, which is associated with the downregulation of the key signaling molecule Ras-Related Protein Rab-27B(RAB27B). Bioinformatic analysis identified a putative forkhead box O1(FOXO1)-binding site in the mouse Rab27b promoter, and chromatin immunoprecipitation supported FOXO1 binding to this region. In mouse proximal tubular cells, high glucose increased FOXO1 phosphorylation and reduced its nuclear accumulation and promoter binding, contributing to RAB27B downregulation and decreased EV secretion (67). Furthermore, calcium ion (Ca2+) influx promotes EV secretion, suggesting a potential link between EV release and plasma membrane repair mechanisms (58, 68).

The cellular status and microenvironment significantly influence the secretion of EVs. During inflammatory states, cells modulate EV secretion through cytokines such as IL-1β and TNF-α to respond to external stimuli (69). Inflammatory microenvironments prompt cells to produce increased numbers of EVs, which may carry molecules that modulate immune responses and play a critical role in disease progression (70, 71). The mechanisms of EV secretion exhibit remarkable heterogeneity across different cell types. For instance, EVs released by tumor cells within the tumor microenvironment can regulate the behavior of surrounding cells, thereby promoting tumor growth and metastasis (72, 73).

### Advanced isolation and purification methods for EVs

Isolation and purification of stem cell-derived exosomes are crucial steps for both basic research and potential clinical applications. Traditional methods, such as ultracentrifugation, are widely used but have drawbacks including low purity, time-consuming procedures, and potential damage to exosomes (74). In recent years, advanced techniques have emerged to address these limitations, offering more efficient and precise methods for obtaining exosomes.

Microfluidic-based separation techniques represent a significant advancement (66). These methods leverage the micro-scale environment to manipulate exosome-containing fluids (75). For instance, the microfluidic pulse-filtration system, developed by integrating micro-channels (resistors), elastic membranes (capacitors), and one-way valves (diodes), can generate micro-liter-scale pulsatile flows that can reciprocate through porous membranes. By cascading filters with different pore sizes, this system can efficiently isolate and purify exosomes from whole-blood samples within 30 minutes, achieving a high recovery rate of up to 80%. The microfluidic approach not only overcomes issues of membrane clogging and particle accumulation but also enables high-throughput processing with minimal sample and reagent consumption, making it highly suitable for analyzing complex biological samples, such as those from diabetes patients (76).

Immunomagnetic bead-based separation is another powerful tool. This method exploits the specific binding between antibodies and antigens on the surface of exosomes (77). Antibodies against common exosomal markers, such as CD9, CD63, or CD81, are conjugated to magnetic beads. When the exosome-containing sample is incubated with these functionalized magnetic beads, the exosomes with the corresponding antigens bind to the beads (78). By applying an external magnetic field, the exosomes can be easily separated from the rest of the sample. Immunomagnetic bead separation offers high specificity, ensuring that the isolated exosomes are of high purity. It also preserves the integrity and biological activity of exosomes better than some traditional methods, as it does not involve harsh physical forces like high-speed centrifugation. This makes it an ideal choice for downstream applications where the biological function of exosomes, such as their immunomodulatory or tissue-repair capabilities in diabetes treatment, needs to be maintained.

Size-exclusion chromatography(SEC) is increasingly utilized for the purification of exosomes. In SEC, the sample containing exosomes is passed through a column filled with porous beads (79). Larger particles, such as cell debris and protein aggregates, are excluded from the pores and elute first, whereas smaller exosomes enter the pores and elute later (80). This process results in the separation of exosomes based on their size. The advantage of SEC is that it can produce exosome preparations with a relatively narrow size distribution, which is crucial for consistent experimental outcomes. Moreover, SEC is a gentle technique that does not expose exosomes to extreme conditions, thereby preserving their native structure and function (81). For example, in studies investigating the role of exosomes in regulating glucose metabolism in diabetes, SEC-purified exosomes can provide more reliable experimental data due to their high purity and intact functionality.

In addition to these methods, techniques such as polymer-based precipitation have also been refined. Polyethylene glycol(PEG) is commonly utilized to precipitate exosomes from biological fluids (79). More recent protocols have optimized the concentration of PEG, incubation time, and temperature to improve precipitation efficiency while reducing the co-precipitation of contaminants. This method is relatively straightforward, cost-effective (82), and can be scaled up for processing large-volume samples, which is advantageous for clinical applications that may require a substantial amount of exosomes for therapies, such as those treating diabetic complications (83). Overall, these advanced methods for isolating and purifying exosomes have significantly enhanced the quality and quantity of exosome preparations, establishing a solid foundation for further research on the role of stem cell-derived exosomes in diabetes and their potential translation into clinical therapies.

## Engineering of MSC-EVs

The engineering of MSC-EVs provides strategies to modify EV cargo and delivery for therapeutic applications. Genetic engineering of donor MSCs is one approach for modifying the molecular cargo of their EVs. For example, Born et al. transfected human bone marrow MSCs with a pCMV-HOTAIR expression plasmid, generating EVs enriched in HOTAIR as confirmed by quantitative reverse transcription PCR. In db/db mice, these EVs improved wound healing compared with unmodified MSC-EVs and increased CD31-positive vessel density compared with the vehicle control. This provides a preclinical example of modifying EV cargo through genetic engineering of the donor MSCs (56) (**Figure 1**).

**Figure 1:**
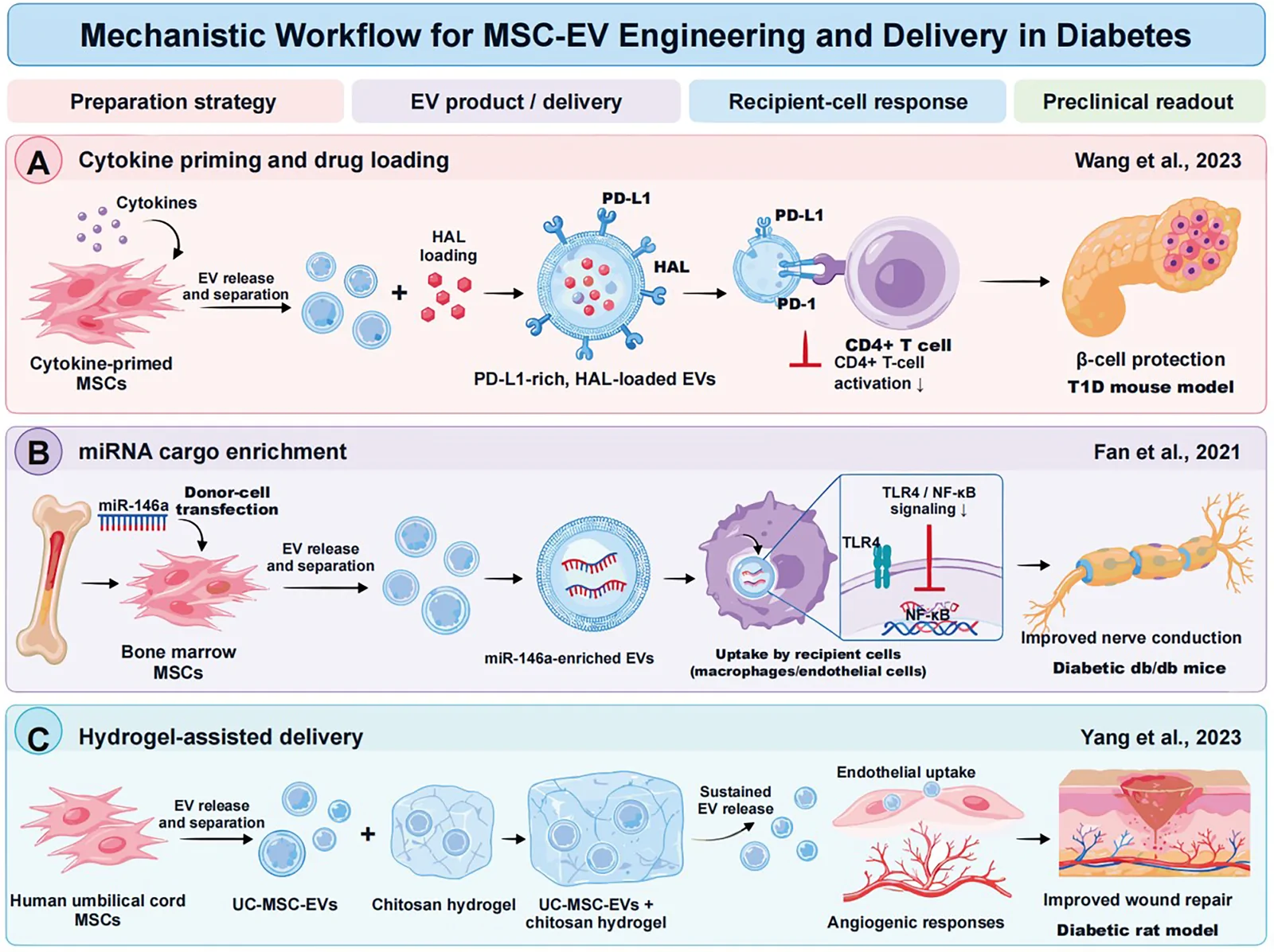
Mechanistic workflow for MSC-EV engineering and delivery in diabetes.The schematic links selected donor-cell modifications or delivery strategies to the resulting EV preparation, recipient-cell response, and disease-model outcome. Parallel examples comprise **(A)** cytokineprimed, HAL-loaded EVs with high PD-L1 expression (19); **(B)** miR-146a-enriched EVs generated through donor-cell transfection (84); and **(C)** chitosan hydrogel–encapsulated EVs (85). Arrows indicate the sequence within each strategy; the examples are not components of a single combined treatment. HAL, hexyl 5-aminolevulinate hydrochloride.

Advancements in drug-loading technologies have further expanded the potential of MSC-EVs for targeted therapy. Loading drugs into EVs can enhance their therapeutic efficacy and improve their targeting capabilities (86, 87). For instance, encapsulating anti-inflammatory drugs, anti-tumor drugs, or other bioactive molecules into MSC-EVs can effectively increase the drug concentration and action time in the target tissue, while reducing drug distribution throughout the body and minimizing side effects. Studies have shown that techniques such as electroporation, ultrasound, or chemical methods can be used to efficiently load drugs into EVs. This not only improves the biocompatibility and stability of EVs but also enhances their ability to target specific cells or tissues (88). The strategies summarized in **Figure 1** distinguish changes to the EV product from changes to its delivery. The strategies summarized in **Figure 1** distinguish changes to the EV product from changes to its delivery. Cytokine-primed MSCs were used to produce PD-L1-rich EVs, which were subsequently loaded with HAL for immunomodulation in a T1D mouse model (**Figure 1A**) (19). For miRNA enrichment, mouse bone marrow MSCs were transfected with a miR-146a oligonucleotide before EV collection, increasing the vesicular miRNA cargo for diabetic neuropathy experiments (**Figure 1B**) (84). For hydrogel-assisted delivery, human umbilical cord MSC-EVs were incorporated into a thermosensitive chitosan hydrogel for sustained release and local application to diabetic rat wounds (**Figure 1C**) (85).

## Mechanisms of MSC-EVs in immunomodulation of diabetes

The MSC sources, animal models, EV doses, administration routes, treatment initiation, dosing schedules, and follow-up periods of representative preclinical studies are summarized in **Supplementary Table S1**. This overview provides the experimental context for interpreting the immunomodulatory and therapeutic findings discussed below.

### Regulation of T cell activity

MSC-EVs can modulate T-cell responses in T1D through multiple mechanisms. In a T1D mouse model, engineered cytokine-primed MSC-EVs with high PD-L1 expression inhibited CD4+ T-cell activation through PD-L1/PD-1 signaling and reduced IFN-γ and TNF-α production (19). These findings provide preclinical evidence for checkpoint-related immunomodulation by this engineered EV preparation. In T1D models, MSC-EVs also promote the expansion of Forkhead Box P3-positive(FOXP3+) regulatory T cells(Tregs), increasing the production of IL-10 and TGF-β to dampen autoimmune attacks (89). The induction of Tregs represents a promising mechanism for achieving long-term immune tolerance, yet challenges remain in maintaining Treg stability and function in the inflamed diabetic microenvironment. Clinically, human clonal MSC-EVs(cMSC-EVs) lower pro-inflammatory cytokines (e.g., IL-1β, IL-17) and elevate Treg populations in streptozotocin-induced diabetic rhesus monkeys, improving glycemic control (90). This non-human primate study bridges preclinical and clinical research, but the translation to human T1D requires validation of dose-dependent effects and long-term safety (**Figure 2**).

**Figure 2:**
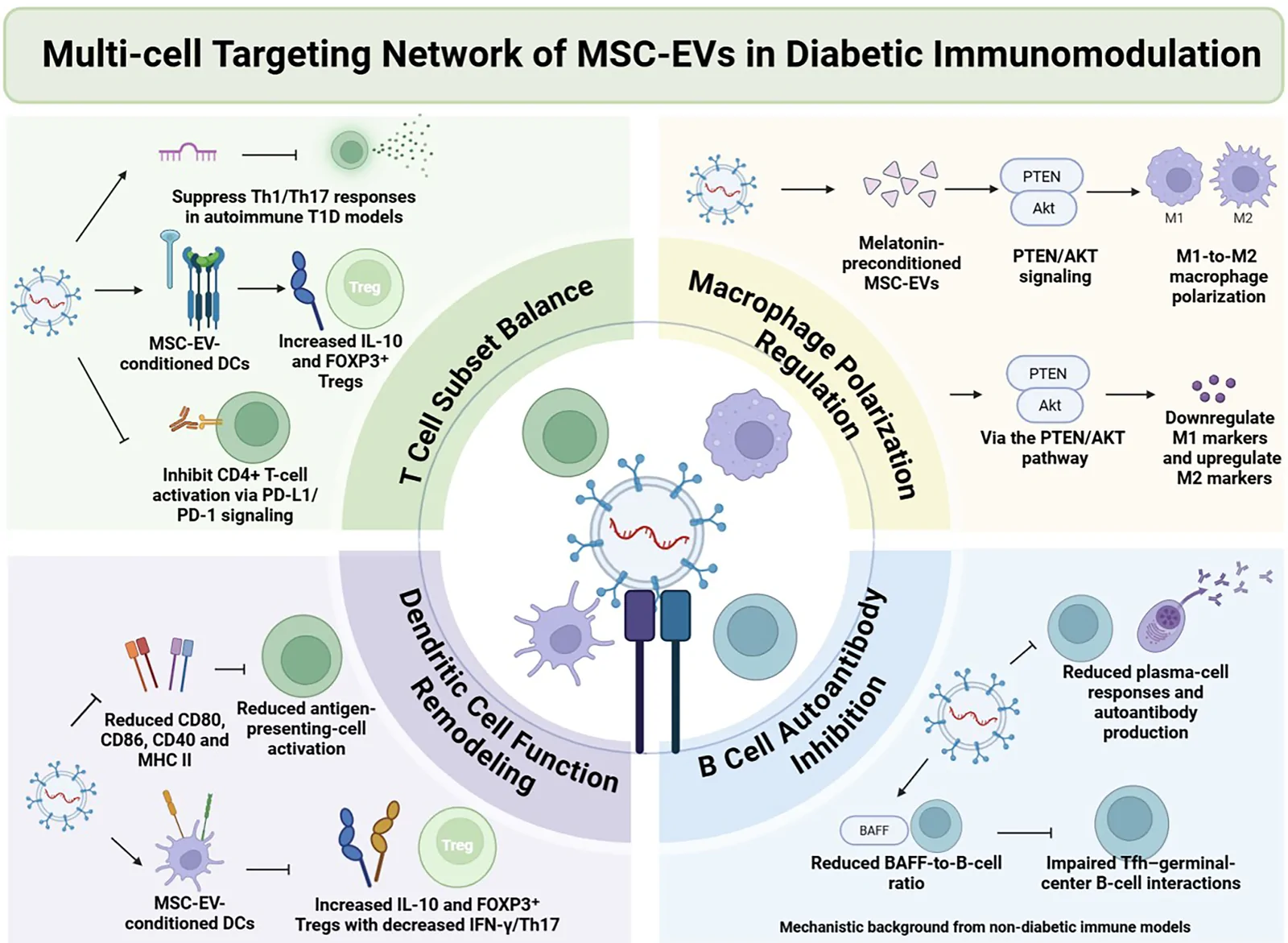
Multi-cell targeting network of MSC-EVs in diabetic immunomodulation. This image illustrates the multi-cell targeting network of MSC-EVs in diabetic immunomodulation, encompassing T cell subset balance (regulating Tregs, inhibiting cytotoxic T cells, etc.), macrophage polarization (promoting M1 to M2 conversion, etc.), dendritic cell function remodeling (downregulating co-stimulatory molecules, etc.), and B cell autoantibody inhibition (inhibiting differentiation and proliferation), all of which collaborate for immunomodulation. This image was created using BioRender.

Th1-associated IFN-γ and Th17-associated IL-17 contribute to inflammatory β-cell injury. In human islets, IL-17 potentiated apoptosis induced by IFN-γ combined with IL-1β or TNF-α (38). MSC-EVs delayed diabetes onset in an adoptive-transfer NOD/scid model, while parallel mixed-lymphocyte experiments showed suppression of Th1/Th17 responses without increased FOXP3+ Tregs (38). Th2 responses have a different role in metabolic inflammation: CD4+ T-cell transfer improved insulin resistance in diet-induced obese Rag1-deficient mice, predominantly through Th2 cells (91). These findings support a protective role for Th2-associated immunity in that setting, but do not establish MSC-EV-induced Th2 differentiation as a mechanism of improved insulin sensitivity.

The pathways summarized in **Figure 2** represent distinct evidence streams rather than a single integrated signaling cascade. In T-cell regulation, PD-L1-high engineered MSC-EVs suppress CD4+ T-cell activation through PD-L1/PD-1 signaling, while MSC-EVs have also been shown to suppress Th1/Th17 responses in an autoimmune T1D model (19, 92). In T1D patient-derived immune-cell cultures, MSC-EV-conditioned DCs increased IL-10 production, reduced IFN-γ and Th17 responses, and increased FOXP3+ Tregs (41). In diabetic wound models, melatonin-preconditioned MSC-EVs promoted an M1-to-M2-associated macrophage shift through phosphatase and tensin homolog(PTEN)/Akt signaling (60). For dendritic-cell regulation, MSC-EVs reduced CD80, CD86, CD40, and major histocompatibility complex(MHC) class II expression on antigen-presenting cells in murine mixed-lymphocyte reactions, whereas the T1D patient-derived model provided complementary functional evidence through altered cytokine and T-cell responses (41, 92). Direct diabetes-specific evidence for MSC-EV-mediated B-cell regulation remains limited. In non-diabetic immune-disease models, MSC-EVs reduced the B-cell-activating factor (BAFF)-to-B-cell ratio and disrupted T follicular helper–germinal-center B-cell interactions, and were also associated with reduced plasma-cell and autoantibody responses (93, 94). These B-cell findings are therefore presented as mechanistic background rather than established pathways in diabetes.

### Modulation of macrophage phenotype

MSC-EVs polarize macrophages from a pro-inflammatory M1 phenotype to an anti-inflammatory M2 phenotype, thereby alleviating islet inflammation. Three-dimensional-cultured human umbilical cord blood MSC-EVs(3D hUCB-MSC-EVs) are enriched with miRNAs, such as miR-124, which promote the expression of M2 markers (CD206, Arginase-1(Arg-1)) and reduce the expression of M1 markers (Inducible Nitric Oxide Synthase(iNOS)) *in vitro (*95). The 3D culture system enhances the therapeutic potency of EVs by mimicking the natural stem cell niche, emphasizing the importance of microenvironment engineering for EV production. However, the clinical scalability of 3D culture systems remains a technical challenge. In diabetic wound models, calcium silicate-stimulated adipose-derived MSC-EVs (CSEVs) incorporated into collagen hydrogels enhance M2 polarization, reduce IL-1β levels, and promote wound healing (96). The combination of EVs with biomaterial scaffolds addresses the short half-life of EVs *in vivo*, but the long-term degradation kinetics of hydrogels and potential foreign body reactions require further investigation. M2 macrophage-derived EVs (M2-EVs) carry miR-23a-3p, which inhibits NLRP3 inflammasome activation in diabetes, restoring the M1/M2 balance around titanium implants (97). This finding highlights the therapeutic potential of EVs derived from polarized immune cells, although the specificity of miR-23a-3p delivery to target tissues needs optimization.

### Regulation of dendritic cell maturation

DCs connect islet antigens to adaptive immunity through MHC class II presentation to CD4+ T cells and MHC class I cross-presentation to CD8+ T cells (37). In murine mixed-lymphocyte reactions, MSC-EVs reduced CD80, CD86, CD40, and MHC class II expression on CD11c+ antigen-presenting cells (92). In an ex vivo study of newly diagnosed T1D, human bone marrow MSC-EV-conditioned DCs pulsed with GAD65 increased IL-10 production and reduced subsequent IFN-γ and Th17 responses while increasing FOXP3+ Tregs (41). These findings support a regulatory effect assessed through both surface markers and functional T-cell responses. Reduced MHC class II expression or costimulation should not, however, be equated with inhibited MHC class I cross-presentation or a uniform indoleamine 2,3-dioxygenase (IDO)/PD-L1-associated tolerogenic phenotype.

In the T1D study, the functional evaluation included cytokine measurements, IFN-γ ELISPOT, and Th17/Treg analyses; increased IL-10 secretion alone does not establish a uniformly IL-10-high DC population (41). In a separate non-diabetic human DC model, MSC-EVs reduced CD80, CD83 and CD38, whereas CD86 and HLA-DR did not change significantly (98). Human islet-derived EVs activated memory T and B cells in peripheral blood mononuclear cells(PBMCs) from patients with T1D. This study provides evidence of islet-EV-associated immune activation, not of its reversal by MSC-EVs (99).

### Regulation of B cells and antibody responses

BAFF supports B-cell survival, and combined blockade of BAFF and a proliferation-inducing ligand(APRIL) reduced insulitis and prevented diabetes in NOD mice (100). Relevant evidence for MSC-EV regulation of humoral immunity comes from other disease settings. In chronic graft-versus-host disease, MSC-EVs reduced the BAFF-to-B-cell ratio and interfered with follicular helper T-cell and germinal-center B-cell interactions (94). Murine bone marrow MSC-EVs were associated with reduced plasmablast differentiation and fewer lymph-node plasmablasts in experimental arthritis (101). In lupus-prone MRL/lpr mice, human umbilical cord MSC-EVs reduced splenic plasma-cell frequencies and serum anti-dsDNA IgG (93). These findings support effects on antibody-secreting-cell responses, but do not establish direct BAFF targeting or reduced islet-specific autoantibody production in T1D.

### Natural killer cells and innate lymphoid cells

In diet-induced obesity, adipose natural killer (NK) cells promote inflammatory macrophage differentiation and insulin resistance through IFN-γ production (102). Human bone marrow MSC-EVs inhibited NK-cell proliferation *in vitro*, with stronger effects following inflammatory priming of the donor MSCs (103). Innate lymphoid cells are functionally heterogeneous: group 2 innate lymphoid cells(ILC2s) can support metabolic homeostasis by promoting white-adipose-tissue beiging (104). Small EVs from human induced pluripotent stem cell-derived MSCs suppressed ILC2 responses in allergic airway models partly through miR-146a-5p delivery (105). This demonstrates EV-mediated ILC modulation, but not a beneficial mechanism in diabetes. Because adipose and airway ILC2s operate in different tissue contexts, the metabolic consequences of MSC-EV-mediated ILC2 suppression require disease-specific assessment.

### Complement regulation

Complement activation provides another link between innate immunity and diabetic tissue injury. Glomerular C4d and C5b-9 deposits were associated with the severity of human diabetic nephropathy (106). A potential EV-mediated regulatory mechanism is the transfer of complement inhibitors. Human umbilical cord MSC-EVs transferred CD59 to retinal astrocytes, reducing C5b-9 membrane attack complex formation and complement-dependent cell death in an experimental setting relevant to neuromyelitis optica spectrum disorders (107). This provides a mechanistic rationale for investigating complement regulation by MSC-EVs, but does not demonstrate therapeutic efficacy against diabetic nephropathy or DR.

### Crosstalk with β cells and immune microenvironment

MSC-EVs directly protect β cells while modulating the immune microenvironment. They deliver miR-191-5p to β cells to activate the PI3K/AKT pathway, enhancing antioxidant capacity and reducing apoptosis (108). The miRNA-mediated protection of β cells from oxidative stress is a key mechanism for preserving insulin secretion, but the specificity of miR-191-5p delivery to β cells versus other cell types requires clarification. As non-MSC evidence, plasma-derived EVs from cold-exposed mice improved insulin sensitivity and reduced β-cell apoptosis in a T2D model, with increased insulin receptor substrate 1 (IRS1) and AKT phosphorylation reported in adipocytes and hepatocytes (109). These are preliminary findings from a single preclinical study, not evidence for cold-preconditioned MSC-EVs.

## Application prospects of MSC-EVs in diabetic complications

### Diabetic foot ulcers and chronic wounds

MSC-EVs have demonstrated remarkable repair functions in diabetic complications, particularly in the context of diabetic foot ulcers(DFUs) and chronic wounds. Reported mechanisms in diabetic wound models include promotion of angiogenesis and regulation of fibroblast function, whereas potential peripheral-nerve repair mechanisms are discussed separately below (110). EVs carry TGF-β, which plays a key role in promoting collagen synthesis and extracellular matrix remodeling, while miR-21 within EVs inhibits the expression of fibrosis-related genes, thus balancing the wound healing process (110, 111). In terms of peripheral nerve repair, adipose MSC-derived EVs have been reported to deliver miR-22-3p to Schwann cells, where miR-22-3p downregulated PTEN and activated AKT/mTOR signaling, thereby promoting Schwann-cell proliferation and migration (112). This evidence was obtained in a peripheral nerve injury model rather than a diabetic foot model and is therefore included as mechanistic background for potential nerve repair in chronic wounds. In a study employing a diabetic mouse model, mitochondrial-derived vesicles(MDVs) isolated from fibroblasts cultured in high glucose conditions were found to exacerbate oxidative stress and mitochondrial dysfunction. Conversely, the inhibition of Sorting Nexin 9(SNX9) restored mitochondrial dynamics and improved wound healing (113). This underscores the critical role of EV-mediated mitochondrial regulation in the pathogenesis of DFUs (**Figure 3**).

**Figure 3:**
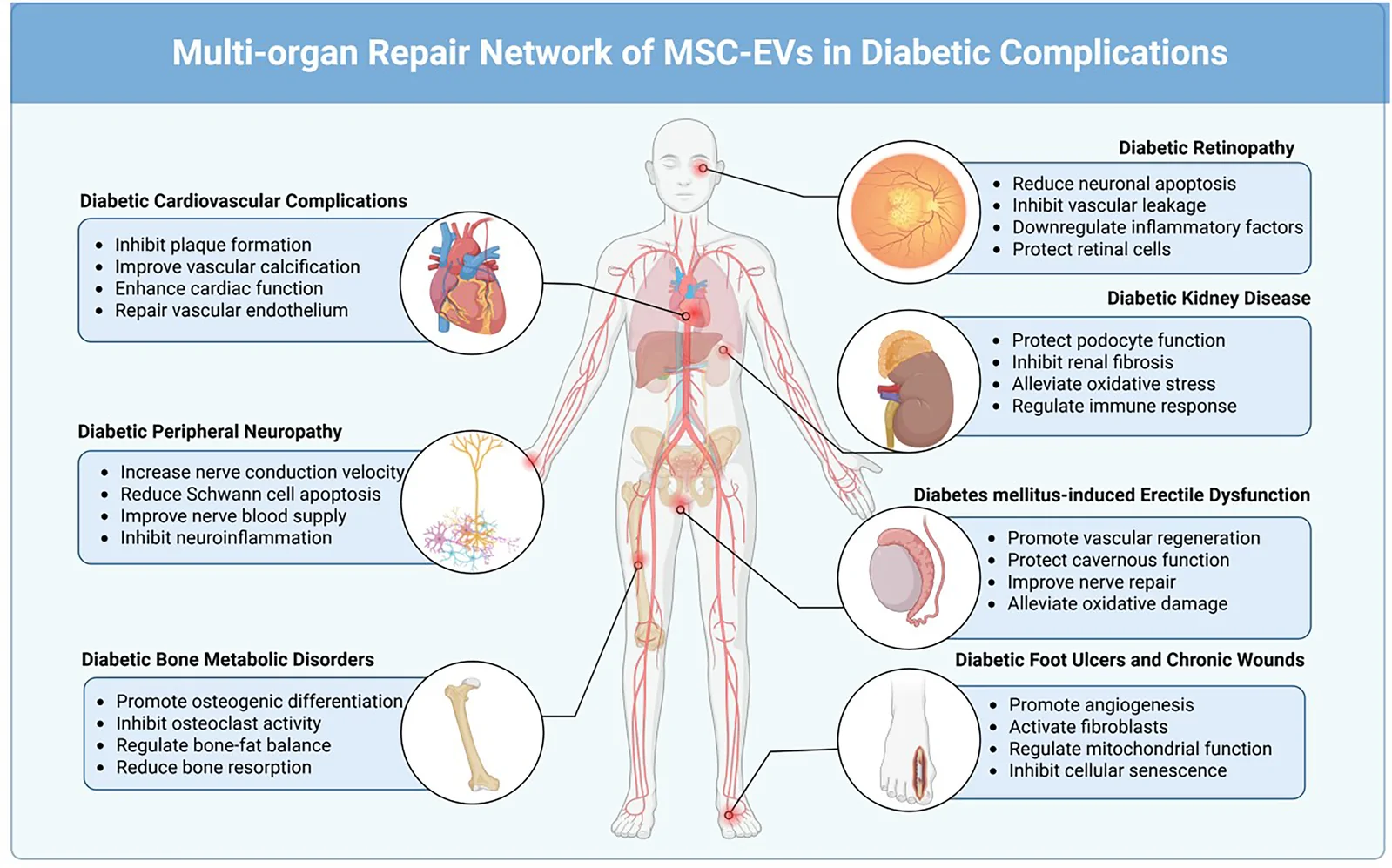
Multi-organ repair network of MSC-EVs in diabetic complications. This image illustrates the multi-organ repair network of MSC-EVs in diabetic complications, encompassing cardiovascular, peripheral nerve, bone metabolism, retina, kidney, erectile function, and foot ulcer systems. These systems collaborate to repair damages caused by diabetic complications. This figure was created using BioRender.

For angiogenesis, a rat model study revealed that aerobic exercise-induced circulating EVs(EXE-EVs) markedly augmented endothelial cell proliferation, migration, and tube formation *in vitro*. Additionally, these EXE-EVs facilitated vascularized skin regeneration in diabetic wound models when administered through decellularized dermal matrix hydrogel (114). In a single study of diabetic rats with full-thickness skin wounds (n = 6 per group), chitosan hydrogel-encapsulated human umbilical cord MSC-EVs improved wound closure and re-epithelialization at day 15 compared with the PBS control (85). These findings represent a model-specific preclinical outcome rather than an established clinical healing rate. Mechanistic studies revealed that MSC-EVs carrying miR-21-5p upregulated VEGF/VEGFR2 expression, thereby stimulating blood vessel sprouting in ischemic diabetic tissues (115).

In the context of cellular senescence, an *in vivo* study conducted on DFU rats demonstrated that adipose-derived MSC-EVs activated the PTEN-induced kinase 1(PINK1)/parkin-mediated mitophagy pathway, which resulted in a reduction of senescence-associated secretory phenotype(SASP) factors and induced apoptosis in senescent fibroblasts (116). For clinical context, an uncontrolled prospective study enrolled 50 patients with diabetes and no-option critical limb-threatening ischemia who received autologous PBMCs. Mean Transcutaneous partial pressure of oxygen (TcPO_2_) increased from 17.2 to 39.1 mmHg during treatment, and ulcer healing with return to walking was reported in 30/50 patients (60%) during a median follow-up of 1.5 years. Circulating small EVs were investigated as biomarkers; these cell-therapy observations do not establish the efficacy of administered MSC-EVs (117).

Excessive neutrophil extracellular trap (NET) formation links innate immune dysregulation to impaired diabetic wound repair. Small EVs from hypoxia-preconditioned human umbilical cord MSCs delivered miR-17-5p to neutrophils, suppressed TLR4/reactive oxygen species (ROS)/mitogen-activated protein kinase (MAPK) signaling, and reduced NET-induced endoplasmic reticulum stress and dysfunction in fibroblasts (118). In another study, NETs promoted endothelial ferroptosis through suppression of PI3K/AKT signaling. Human umbilical cord MSC-EV treatment restored neutrophil mitochondrial function, reduced NET formation, and improved angiogenesis and wound healing in diabetic mice (119). These findings identify neutrophil regulation as an additional mechanism through which MSC-EVs can protect the cells involved in tissue repair.

The development of biomaterial-based carrier technologies, such as hydrogels, has further advanced the clinical application of EVs. Hydrogels serve as an ideal vehicle for EVs, offering several advantages: they protect EVs from degradation, prolong their residence time at the wound site, and enable controlled release (120, 121). For instance, 3D-printed hydrogels can be precisely tailored to fit the shape of the wound, ensuring uniform distribution of EVs and sustained release throughout the healing process (122, 123). Furthermore, hydrogels themselves can modulate the local inflammatory response, creating a supportive microenvironment that enhances the therapeutic effects of EVs (124). In an investigation, a modified graphene oxide-based hydrogel was shown to enhance the secretion of MSC-EVs containing miR-21. These vesicles targeted the Plasmacytoma Variant Translocation 1(PVT1)/PTEN/IL-17 axis, reducing the expression of pro-inflammatory cytokines such as TNF-α and IL-17, and thereby promoting wound closure in diabetic mouse models (125). This integrated approach, which combines EVs with biomaterial carriers, represents a significant step forward in translating EV-based therapies for DFUs and other chronic wounds, holding great promise for improving clinical outcomes in diabetic patients.

### Diabetic retinopathy

MSC-EVs are being investigated as a preclinical therapeutic approach for DR, a major microvascular complication of diabetes. A global systematic review and meta-analysis estimated the prevalence of DR at 22.27% among people with diabetes (126). DR is characterized by blood-retinal barrier(BRB) dysfunction, retinal hypoperfusion or ischemia, and pathological neovascularization, which can ultimately contribute to vision loss (127). Preclinical studies have reported anti-inflammatory, vascular, and neuroprotective effects of MSC-EVs in experimental retinal injury and diabetic models (128, 129).

In a rat model of retinal ischemia-reperfusion, the intravitreal administration of MSC-EVs significantly reduced neuronal apoptosis and microglial activation (128). Fluorescent tracing demonstrated that EVs were internalized by retinal ganglion cells and microglia within 24 hours, followed by an upregulation of anti-apoptotic proteins, such (as B-cell lymphoma 2(Bcl-2), and a suppression of pro-inflammatory cytokines, (including TNF-α and IL-1β. This dual activity is crucial in combating the neurodegeneration associated with DR, where persistent hyperglycemia induces oxidative stress and glial cell dysfunction, resulting in the progressive loss of retinal neurons.

Bevacizumab-loaded MSC-sEVs have been evaluated as a sustained intraocular anti-VEGF delivery strategy in a DR model. In diabetic mice, this formulation prolonged anti-VEGF activity, reduced the required frequency of intravitreal administration, maintained vitreous clarity for more than eight weeks, and decreased VEGF-associated leukostasis and hard-exudate formation compared with free bevacizumab (130). These findings support sustained EV-mediated anti-VEGF delivery as a potential strategy to reduce the burden of repeated intravitreal administration. Compared to traditional stem cell therapy, which is constrained by immune rejection, stem cell-derived EVs present an emerging therapeutic approach that has shown safety and efficacy. EVs circumvent the immunological risks associated with cell transplantation and demonstrate exceptional biocompatibility, making them an ideal alternative—especially for the long-term management of DR patients (131).

Molecularly, human umbilical cord MSC-EVs carrying miR-30c-5p target the inflammatory cascade that underlies DR progression. In streptozotocin-induced diabetic rats, these EVs inhibited the Phospholipase C Gamma 1(PLCG1)/Nuclear Factor kappa-light-chain-enhancer of Activated B cells(NF-κB) pathway in retinal endothelial cells, thereby reducing vascular permeability and leukocyte infiltration (129). The miR-30c-5p cargo directly targets PLCG1, a key mediator of protein kinase C(PKC) activation, disrupting the pro-inflammatory signaling that drives BRB breakdown. This mechanism is particularly relevant in DR, where chronic inflammation and oxidative stress synergistically promote endothelial dysfunction and pericyte loss, early events in DR pathogenesis.

### Diabetic kidney disease

Diabetic kidney disease(DKD) is characterized by chronic tubulointerstitial fibrosis and glomerulosclerosis, which ultimately leads to end-stage renal disease(ESRD) necessitating dialysis or kidney transplantation. Current evidence-based management includes glycemic and blood-pressure control, renin–angiotensin system blockade when indicated, and, in appropriate patients, sodium-glucose cotransporter 2 inhibitors, glucagon-like peptide-1(GLP-1) receptor agonists, and the nonsteroidal mineralocorticoid receptor antagonist finerenone; nevertheless, substantial residual risk of CKD progression remains (132).

MSC-EVs have emerged as promising candidates for the treatment of DKD. These vesicles mitigate renal injury in DKD by modulating podocyte function and inhibiting fibrosis. In high-glucose-exposed mouse podocytes, EVs from mouse adipose-derived MSCs delivered miR-26a-5p, which targeted TLR4 and reduced NF-κB signaling, VEGFA expression, and apoptosis. Inhibition of miR-26a-5p attenuated these protective effects. The study also reported renal protection in db/db mice, supporting this pathway as a preclinical strategy for limiting podocyte injury in DKD (54). More recently, bone marrow MSC-derived EVs were reported to deliver miR-143-3p to podocytes, where miR-143-3p directly targeted Bcl-2 and relieved Bcl-2-mediated inhibition of Beclin1-dependent autophagy (133). In high-glucose-treated podocytes and diabetic mice, this miR-143-3p/Bcl-2/Beclin1 axis was associated with enhanced autophagy, preserved podocin expression, and reduced renal injury. Likewise, bone marrow MSC-EVs, enriched with miR-15b-5p, protected podocytes by downregulating Pyruvate Dehydrogenase Kinase 4(PDK4), thereby inhibiting the VEGF/PDK4 axis and reducing reactive oxygen species production (134). In streptozotocin-induced diabetic mice, intravenous administration of liver stem-like cell-derived EVs led to decreased albuminuria and renal fibrosis, along with reduced expression of collagen I and fibronectin (135). Transcriptomic analysis indicated that these EVs modulated Wnt/β-catenin signaling and inhibited tubular epithelial-to-mesenchymal transition. These findings collectively highlight the multi-target therapeutic potential of MSC-EVs in DKD, as they simultaneously address podocyte injury, oxidative stress, and fibrotic remodeling. The consistency of anti-fibrotic effects across different MSC-EV sources (adipose, bone marrow, liver) suggests conserved mechanisms, while the specificity of miRNA cargo (e.g., miR-26a-5p, miR-15b-5p) underscores the precision of their regulatory roles. Modulation of Wnt/β-catenin signaling may therefore contribute to the anti-fibrotic effects of these EV preparations in DKD.

MSC-EVs also exert protective effects against DKD by regulating inflammatory responses and immune cell behavior. In high-glucose-exposed podocytes, MSC-derived EV treatment suppressed activation of the NOD2 signaling pathway and reduced the downstream phosphorylation of RIP2 and NF-κB p65, accompanied by lower TNF-α, IL-6, IL-1β, and IL-18 expression and reduced apoptosis (136). In diabetic mice, MSC-derived EVs likewise attenuated renal inflammation, urinary albumin excretion, and renal injury while suppressing NOD2 pathway activation. Co-culture experiments demonstrated that macrophage-derived EVs with elevated miR-21-5p promoted podocyte pyroptosis through A20 inhibition, whereas MSC-EVs counteracted this effect by delivering anti-inflammatory miRNAs (137). In db/db mice, MSC-EVs decreased renal infiltration of CD68+ macrophages and downregulated pro-inflammatory cytokines by suppressing NF-κB signaling (138). Furthermore, urinary EVs from DKD patients exhibited altered miRNA profiles, with upregulation of miR-192 and miR-375 correlating with the severity of albuminuria (139, 140). Plasma EVs from DKD patients showed increased levels of uracil and sphingosine 1-phosphate, which were identified as early biomarkers through liquid chromatography-mass spectrometry (141). The ability of MSC-EVs to counteract pro-inflammatory EVs from macrophages underscores their immunomodulatory dominance in the pathogenesis of DKD. The reduction of renal macrophage infiltration in preclinical models suggests a potential strategy for halting inflammation-driven tissue damage. Notably, the discovery of patient-derived EV biomarkers connects basic research with clinical translation, indicating that MSC-EVs serve not only as therapeutics but also inform diagnostic strategies. The metabolic reprogramming suggested by the elevation of sphingosine 1-phosphate further establishes EVs as central hubs for integrating immune-metabolic crosstalk in DKD.

The therapeutic potential of MSC-EVs in DKD is further supported by their ability to restore renal progenitor cell function. Flow cytometric analysis revealed that CD133+ urinary EVs, derived from renal progenitor cells, were significantly reduced in DKD patients with albuminuria, reflecting impaired regenerative capacity (142). *In vitro*, glucose-overloaded renal progenitor cells released EVs with decreased CD133 expression, which was reversed by MSC-EV co-treatment. Additionally, MSC-EVs improved renal function in Akita mice by inhibiting hedgehog interacting protein (Hhip)-induced tubular senescence, as demonstrated by reduced extracellular vesicle release and β2-microglobulin-mediated fibrosis (143). Proteomic analysis of MSC-EVs identified proteins involved in mitochondrial function and antioxidant defense, which contributed to mitigating oxidative stress in renal tubular cells (138). By rescuing CD133+ EVs and reversing progenitor cell dysfunction, MSC-EVs address a critical deficit in DKD: the failure of renal repair mechanisms. The targeting of Hhip-induced senescence highlights a novel anti-aging strategy for preserving tubular integrity, while proteomic identification of mitochondrial proteins (e.g., antioxidant enzymes) mechanistically links MSC-EVs to cellular bioenergetics restoration. These findings collectively suggest that MSC-EVs act as “regenerative messengers,” simultaneously enhancing progenitor cell activity and protecting existing renal parenchyma from oxidative/senescence damage. The translational value of mitochondrial proteomics may guide the design of next-generation MSC-EV-based therapies for DKD.

### Diabetic cardiovascular complications

In diabetes-exacerbated atherosclerosis, bone marrow mesenchymal stem cell-derived EVs(BMSC-EVs) attenuated atherosclerotic progression through adenosine monophosphate-activated protein kinase/mammalian target of rapamycin(AMPK/mTOR)-regulated autophagy and macrophage polarization in apolipoprotein E-deficient(ApoE−/−) mice (144). In diabetic ApoE−/− mouse models, treatment with BMSC-EVs has led to a significant reduction in blood glucose levels, Low-Density Lipoprotein(LDL) levels, and aortic plaque area, accompanied by an increase in collagen content. The underlying mechanism is believed to involve the promotion of autophagosome/autolysosome formation and a reduction in the proportion of M1 macrophages (144). Furthermore, a composite hydrogel consisting of BMSC-EVs and a peptide derived from histone deacetylase 7 has been shown to synergistically suppress inflammation and promote angiogenesis, thereby accelerating diabetic wound healing and presenting a combined therapeutic approach for diabetic vascular complications (145).

Adipose-derived mesenchymal stem cell EVs (ADSC-EVs) play a pivotal role in mitigating diabetes-related vascular complications. In diabetic atherosclerosis models, ADSC-EVs suppress macrophage activation through the transfer of anti-inflammatory miRNAs, such as miR-146a. This reduces inflammatory cell infiltration in plaques and promotes endothelial progenitor cell migration, thereby repairing damaged vascular endothelium (146). Notably, phosphatases Protein Tyrosine Phosphatase 1B(PTP1B) and Protein Phosphatase 2A(PP2A) present in ADSC-EVs enter cardiomyocytes via the circulatory system. They inhibit the dephosphorylation of IRS1-Akt signaling, thereby enhancing insulin sensitivity in diabetic cardiomyocytes. This process also alleviates myocardial fibrosis and systolic dysfunction (147).

Beyond mesenchymal stem cells, EVs derived from cardiomyocytes exhibit protective effects in diabetic cardiomyopathy. For example, exosomes from ticagrelor-pretreated cardiomyocytes inhibit hyperglycemia-induced cardiomyocyte apoptosis by alleviating oxidative and endoplasmic reticulum stress, while also promoting endothelial cell migration and tube formation to enhance cardiac function (148). This discovery not only connects fundamental research on EV biology with clinical pharmacology but also opens the door to personalized EV-based therapies. In these therapies, drug-preconditioned EVs could act as “smart vectors” for comprehensive cardioprotection. Nevertheless, challenges persist in scaling up the production of ticagrelor-primed EVs and in establishing their long-term safety profile. These issues necessitate further translational research to unlock their full clinical potential.

### Diabetic bone metabolic disorders

DOP develops from a complex interplay of metabolic dysregulation, inflammation, and impaired bone remodeling. Hyperglycemia-driven mechanisms disrupt the balance between osteoblasts and osteoclasts. Elevated glucose levels lead to the accumulation of AGEs, which hinder osteoblast differentiation and increase osteoclast activity by upregulating Receptor Activator of Nuclear Factor-κB Ligand(RANKL). Concurrently, oxidative stress, induced by high glucose levels, activates the NLRP3 inflammasome in osteoclasts, thereby exacerbating bone resorption (149). Insulin resistance impairs the PI3K/Akt pathway, which results in reduced osteoblast proliferation and collagen synthesis. Han et al. directly investigated the role of BMSC-derived EVs in diabetic bone-fat imbalance (150). EVs from normal mouse BMSCs enhanced osteogenesis and suppressed adipogenesis, whereas these effects were attenuated in EVs from diabetic BMSCs; elevated EV-associated miR-221 contributed to reduced osteogenesis and increased adipogenesis. Clinical studies indicate that T2D is linked to a higher fracture risk, even when bone mineral density(BMD) is normal or elevated, underscoring the significance of bone quality over quantity (151, 152).

MSC-EVs alleviate DOP through various mechanisms. ADSC-EVs suppress NLRP3 inflammasome activation in osteoclasts by transferring miR-26a-5p, which decreases IL-1β secretion and restores bone mass in streptozotocin-induced rats (149). BMSC-EVs enhance osteogenesis by delivering miR-140-3p to target Plxnb1, a receptor that inhibits osteocyte differentiation, thereby promoting bone regeneration in diabetic rats (53). Engineered BMSC-EVs, loaded on Gold Magnetic Nanoparticles(GMNPs), improve the delivery of miR-150-5p to osteoblasts, activating the Wnt/β-catenin signaling pathway via MMP14 inhibition and accelerating bone formation (22). Urine-derived stem cell-EVs (USCs-EVs) deliver miR-26a-5p to repress Histone Deacetylase 4(HDAC4), activating the Hypoxia-Inducible Factor 1 Alpha(HIF-1α)/VEGFA pathway to promote osteoblast differentiation and inhibit osteoclast activity (153). Notably, H2O2-preconditioned ADSC-EVs enhance the activation of the Nuclear Factor Erythroid 2-Related Factor 2(NRF2)/Heme Oxygenase-1(HO-1) pathway in diabetic BMSCs, reducing cellular senescence and restoring osteogenic capacity (154).

Translational challenges encompass the heterogeneity of MSC-EVs, with diabetic BMSC-EVs exhibiting reduced osteogenic potential due to an adipogenic shift mediated by miR-221 (150). Additionally, systemic EVs have low bone targeting efficiency, which can be mitigated by delivery systems based on GMNPs (22, 155). Clinical applications must also address the pro-fibrotic potential of certain EV cargoes and establish standardized isolation protocols. Future studies may concentrate on combinatorial strategies, such as MSC-EVs combined with anti-inflammatory peptides or biomimetic scaffolds, to enhance therapeutic efficacy while minimizing off-target effects (145). In summary, MSC-EVs represent a promising cell-free therapy for DOP, capitalizing on their immunomodulatory and regenerative properties to tackle both metabolic and skeletal dysfunctions.

### Diabetic peripheral neuropathy

Diabetic peripheral neuropathy(DPN) pathogenesis is primarily driven by hyperglycemia-induced oxidative stress, inflammatory cascades, and neurovascular dysfunction. Hyperglycemia leads to the formation of AGEs, which activate the TLR4/NF-κB pathway, thereby promoting the release of pro-inflammatory cytokines such as IL-1β and TNF-α, and exacerbating neuroinflammation (59, 84). Mitochondrial dysfunction and oxidative stress are prominent pathological features of diabetic peripheral neuropathy; in db/db mice, these abnormalities were accompanied by impaired mitochondrial homeostasis and neurological dysfunction (20). Vascular endothelial dysfunction diminishes nitric oxide synthesis and thickens capillary basement membranes, fostering an ischemic microenvironment (59). These interconnected mechanisms constitute a “metabolic-inflammation-vascular” triad, highlighting the necessity for disease-modifying therapies that extend beyond mere symptomatic relief.

MSC-EVs rewire DPN pathology by delivering functional cargo. Fan et al. generated miR-146a-enriched EVs by transfecting mouse bone marrow MSCs. These EVs reduced Interleukin-1 receptor-associated kinase 1(IRAK1) and Tumor necrosis factor receptor-associated factor 6(TRAF6) expression in sciatic nerves and suppressed TLR4/NF-κB-associated inflammatory responses in macrophages and endothelial cells. In db/db mice, treatment improved nerve conduction and neurovascular perfusion, linking miR-146a delivery to protection against inflammatory neurovascular injury (84). In one study using 20-week-old db/db mice with diabetic peripheral neuropathy, EVs from SIRT1-overexpressing bone marrow MSCs increased mitochondrial membrane potential and improved nerve conduction relative to control MSC-EVs (20). These findings support a potential mitochondrial protective effect in this model, without establishing a generalizable percentage of functional recovery. GMNP-EVs enrich in sciatic nerves, with miR-130a-3p activating NRF2/HIF1α to increase vascular density by 2.3-fold, highlighting engineering strategies to improve neurotargeting (156). A meta-analysis of EVs from multiple cellular sources found improved motor nerve conduction velocity across seven studies (standardized mean difference [SMD] 4.71, 95% CI 2.18–7.25; I² = 91.8%) and improved plantar skin blood-flow perfusion across two studies (SMD 2.84, 95% CI 0.89–4.80; I² = 74.9%) (157). The substantial heterogeneity and inclusion of non-MSC EVs limit interpretation of these pooled estimates as MSC-EV-specific treatment effects.

Clinical translation encounters challenges: diabetic patient-derived MSC-EVs exhibit reduction in neuroregenerative capacity due to the upregulation of miR-221 (150), necessitating the screening of donors. Encapsulation of MSC-EVs in chitosan hydrogel extends their local retention threefold (145). Future research should prioritize the design of dual-target EVs and multicenter trials to establish standardized production processes for clinical translation.

### Diabetes mellitus-induced erectile dysfunction

Diabetes mellitus-induced erectile dysfunction(DMED) arises from complex pathophysiological processes involving vascular, neural, and structural impairments. Chronic hyperglycemia drives oxidative stress, leading to endothelial dysfunction characterized by reduced nitric oxide(NO) bioavailability—a key mediator of cavernosal smooth muscle relaxation (158). This is compounded by diabetic neuropathy, which disrupts cavernous nerve signaling, and fibrosis of the corpus cavernosum, where smooth muscle loss and collagen deposition impair erectile function (158, 159). Additionally, hyperglycemia-induced phenotypic switching of corpus cavernosum smooth muscle cells(ccSMCs) from a contractile to a synthetic state further compromises tissue elasticity. Collectively, these mechanisms create a multifaceted barrier to effective erection, often refractory to conventional therapies like phosphodiesterase 5 inhibitors (160).

A meta-analysis reporting 20 studies involving 324 rats included eight studies using diabetic models (161). Across the combined erectile dysfunction models, EV treatment increased the intracavernosal pressure/mean arterial pressure ratio (SMD 4.19, 95% CI 3.31–5.08; I² = 77%). This pooled estimate is not specific to diabetes or MSC-EVs, and nonsignificant subgroup differences do not establish equivalent efficacy across EV sources or disease models. Mechanistically, MSC-EVs enriched with miR-200a-3p reversed erectile dysfunction in streptozotocin-induced diabetic rats by targeting Keap1, thereby activating the NRF2/HO-1 antioxidant pathway to reduce oxidative stress and ccSMC apoptosis (158). This study highlighted the potential of miRNA engineering to enhance EV efficacy, as miR-200a-3p-enriched EVs outperformed unmodified EVs in restoring smooth muscle content and reducing fibrosis. Notably, embryonic stem cell-derived EV-mimetic nanovesicles (ESC-NVs) achieved near-complete restoration of erectile function in diabetic mice by promoting angiogenesis and neural regeneration, surpassing the efficacy of stem cell transplantation alone, suggesting that EVs may offer superior bioactivity by concentrating pro-regenerative cargo (159).

Beyond MSC-EVs, other stem cell-derived EVs have demonstrated therapeutic potential through distinct mechanisms. USC-EVs ameliorated DMED in type 2 diabetic rats by delivering proangiogenic miRNAs (e.g., miR-21-5p, let-7 family), enhancing endothelial cell proliferation, migration, and tube formation under high glucose conditions (162). When combined with hyaluronic acid(HA) for sustained local delivery, USC-EVs further improved angiogenesis and smooth muscle regeneration, with multiple doses yielding superior outcomes—supporting the need for optimized dosing strategies (163). Similarly, pericyte-derived EVs enriched with miR-148a-3p promoted cavernous neurovascular regeneration in diabetic mice by inhibiting PDK4, a key regulator of metabolic stress, underscoring the tissue-specific utility of EVs in targeting neurovascular damage (164).

## Therapeutic and repair mechanisms of MSC-EVs in diabetic β-cell injury

### Therapeutic mechanisms of MSC-EVs in T1D β-cell injury

MSC-EVs have shown immunoregulatory activity in preclinical T1D models. In an autoimmune T1D model, MSC-EV treatment suppressed Th1/Th17 responses, whereas engineered PD-L1-high MSC-EVs inhibited CD4+ T-cell activation through PD-L1/PD-1 signaling (19, 92). These findings support immunomodulation as one potential route through which MSC-EV preparations may protect β-cell function in T1D. In an STZ-induced T1D rat model, EVs from menstrual blood-derived MSCs promoted β-islet regeneration and insulin production, with pancreatic and duodenal homeobox 1(PDX1) implicated in the regenerative response (165). Separately, human MSC-derived EVs increased PI3K and BCL-2 expression while reducing BCL-2-associated death promoter(BAD) and BCL-2-associated X protein(BAX) expression and apoptosis in isolated mouse islets; because this evidence was obtained in an ex vivo islet model rather than an *in vivo* T1D intervention, it is presented here as supportive evidence for PI3K/BCL-2-associated islet survival rather than as a directly established T1D-specific PI3K/Akt pathway (166). The DC-mediated regulation of autoreactive T-cell responses relevant to β-cell protection is discussed in “Regulation of Dendritic Cell Maturation (41).

### Repair mechanisms of MSC-EVs in T2D β-cell metabolic dysregulation

In T2D, β-cell metabolic dysfunction is primarily caused by insulin resistance, lipotoxicity, and oxidative stress. MSC-EVs alleviate β-cell dysfunction by enhancing insulin sensitivity and modulating metabolic signaling pathways.

MSC-EVs also play a role in countering oxidative stress-related β-cell damage in T2D. In an STZ-induced INS-1 oxidative-damage model, human umbilical cord MSC-EVs enriched in miR-191-5p targeted death-associated protein kinase 1 (DAPK1) and activated PI3K/AKT signaling, with reduced ROS production and apoptosis (108). In a separate glucose-stimulated insulin-secretion assay within the same study, MSC-EV treatment increased insulin secretion from INS-1 cells under high-glucose conditions. Additional mechanisms summarized in **Figure 4** are supported by distinct experimental settings. In hypoxic βTC-6 cells, human umbilical cord MSC-EVs enriched in miR-21 reduced endoplasmic reticulum stress-related proteins and p38 MAPK phosphorylation, thereby improving β-cell survival (167). In high-fat diet/streptozotocin(HFD/STZ)-induced T2D rats, human umbilical cord MSC-EVs restored IRS1/AKT signaling, promoted glucose transporter 4(GLUT4) expression and membrane translocation in skeletal muscle, and increased hepatic glycogen storage, supporting improved peripheral insulin responsiveness and glucose homeostasis (21). In aging-associated insulin resistance, elevated miR-29b-3p in bone marrow MSC-EVs targeted SIRT1 and impaired insulin sensitivity, whereas reducing exosomal miR-29b-3p improved insulin resistance (168). By integrating these anti-inflammatory, metabolic-regulating, and antioxidant mechanisms, MSC-EVs offer a multifaceted approach to preserving β-cell mass and function in T2D.

**Figure 4:**
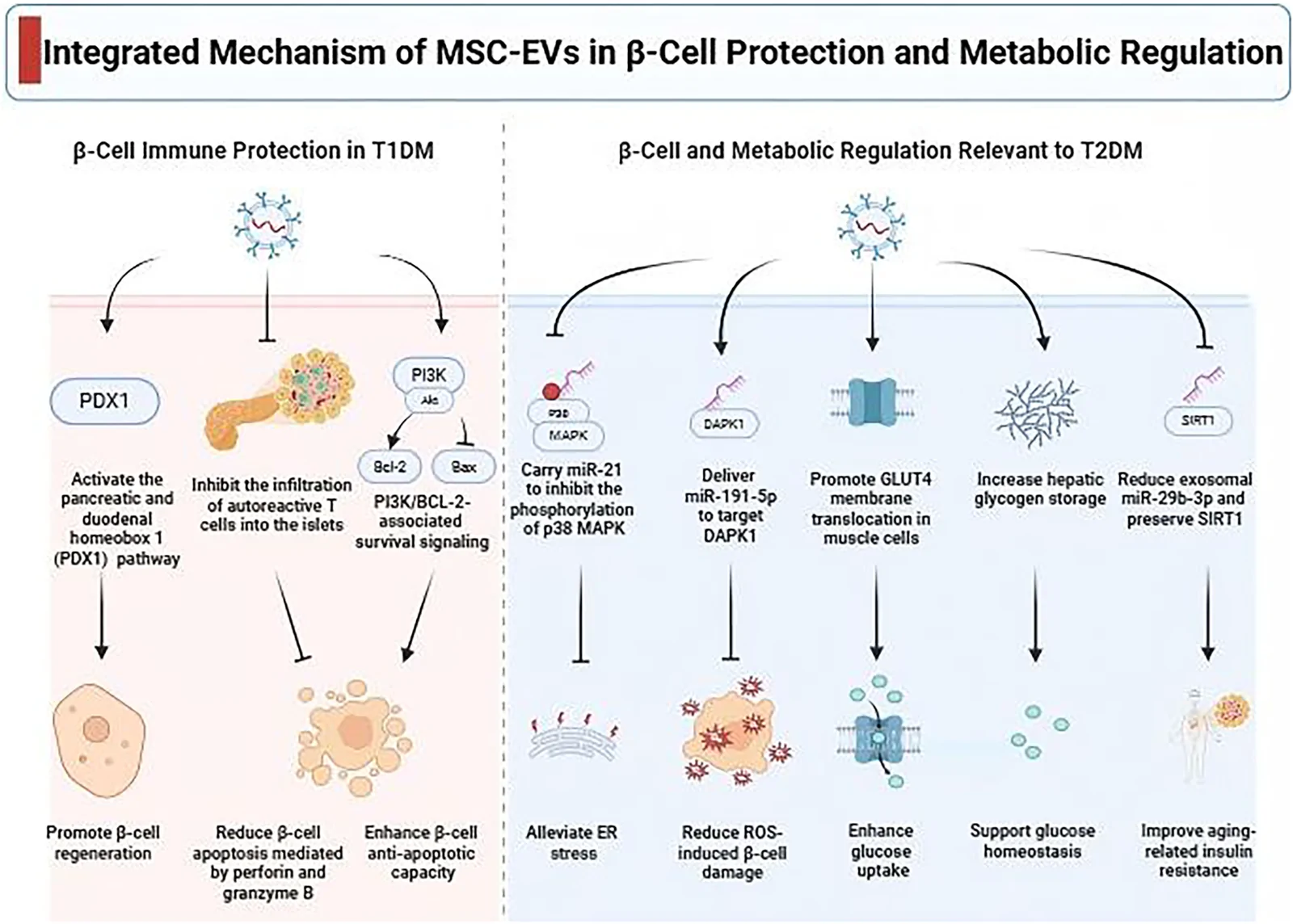
Integrated mechanism of MSC-EVs in β-cell protection and metabolic regulation. This image illustrates the integrated mechanism of MSC-EVs in β-cell protection and metabolic regulation. In T1DM, they promote β-cell regeneration and anti-apoptosis via immune protection. In T2DM, they alleviate endoplasmic reticulum stress, promote proliferation and secretion through metabolic protection, regulate glucose uptake and production, and improve insulin resistance. This image was created using BioRender.

### Clinical evidence and registered trials

Registered investigations of MSC-EVs in diabetes and its complications are summarized in **Table 2**, including trial identifiers, phases or registered study categories, sponsors, interventions, endpoints, status dates, and available results. The identifiable records address T1D, T2D, diabetic foot ulcers, and diabetes-associated erectile dysfunction. Registration and planned outcome measures are distinguished from reported clinical results.

**Table 2:** Registered clinical studies of MSC-EVs in diabetes and its complications.

Trial ID/condition	Phase/stage	Sponsor	Intervention/route	Endpoints	Status/record date	Available results
NCT02138331 (1) T1D	2/3 in phase field	General Committee of Teaching Hospitals and Institutes, Egypt	Cord-blood MSC-EV fractions described as exosomes and microvesicles; IV administrations 7 days apart.	Primary: total daily insulin dose at 3 months. Other listed measures: β-cell mass and HbA1c.	Unknown; last known: enrolling by invitation. Record: 14 May 2014.	No posted results in the retrieved record; no trial-specific clinical results publication identified.
NCT07693036 (2) T2D (HOPE-T2DM)	1/2 (b)	Peking University First Hospital, China; collaborator: ECHO Biotech	hUC-MSC-EVs (GoldenExo/NATX); IV weekly for 8 doses. Dose escalation, then randomized EV-free-buffer-controlled stage.	Primary: HbA1c change at week 8. Selected secondary: CGM time in range, insulin/C-peptide and longer-term metabolic measures. Safety monitored.	Recruiting. Record: 9 July 2026.	No results posted in the retrieved record; no trial-specific clinical results publication identified.
NCT06812637 (3,7) DFU	1 (b)	Kafrelsheikh University, Egypt	Topical WJ-MSC-EV gel plus SOC; comparators: SOC alone and vehicle gel plus SOC. Administration frequency differs within the publication (c).	Publication: ulcer-area reduction.	Completed in registry-derived copy (b). Record date in copy: 29 April 2025.	2025 report: 110 randomized, 85 analyzed; mean healing time reported as 6 vs 20 weeks. Attrition and internal reporting discrepancies limit interpretation (c).
ChiCTR2500103555 (4) T2D with DFU	Exploratory/pilot study (asregistered)	Chinese PLA General Hospital, China	Local subcutaneous hUC-MSC exosome administration versus conventional treatment; multicenter randomized study.	Wound healing rate, overall response measure and positive sweating-test rate. Endpoint time points not specified in the retrieved record.	Not yet recruiting in cited version. Version 1.0: 30 May 2025.	No outcome results identified in the retrieved registry information or trial-ID-linked publications.
ChiCTR2600121779 (5) T2D with DFU	Exploratory/pilot study (as registered)	The Second Affiliated Hospital of Zhejiang University School of Medicine, China	Topical MSC exosome suspension after debridement, then hydrogel cover; 5×10¹^0^, 1×10¹¹ or 2×10¹¹ particles. Tissue source not specified.	Primary: 28-day DLT, treatment-related AEs/SAEs, MTD/RP2D. Secondary: 12-week closure, 4-week area reduction, healing time and amputation.	Not yet recruiting in cited record. Updated: 2 April 2026.	No outcome results identified in the retrieved registry information or trial-ID-linked publications.
NCT07480161 (6) Diabetic ED	2/3 (b)	Ankara City Hospital Bilkent, Türkiye	Separate arms: single intracavernosal UC-MSC exosomes (75 μg), live UC-MSCs (5×10^6^ cells), or saline. Not combined cell–EV therapy.	Primary: IIEF-5 and EHS at months 1, 3, 6 and 12. Secondary: penile Doppler measures and adverse events.	Recruiting. Record: 18 March 2026.	No results posted in the retrieved record; no trial-specific clinical results publication identified.

MSC, mesenchymal stem/stromal cell; EV, extracellular vesicle; UC, umbilical cord; WJ, Wharton’s jelly; T1D/T2D, type 1/type 2 diabetes; DFU, diabetic foot ulcer; ED, erectile dysfunction; IV, intravenous; SOC, standard of care; CGM, continuous glucose monitoring; DLT, dose-limiting toxicity; AE/SAE, adverse/serious adverse event; MTD, maximum tolerated dose; RP2D, recommended phase 2 dose; IIEF-5, five-item International Index of Erectile Function; EHS, Erection Hardness Score. Registry product names do not independently establish EV biogenesis or marketing approval.

A randomized trial of topical Wharton’s jelly MSC-EVs reported improved diabetic foot ulcer healing, but only 85 of 110 randomized participants were included in the final analysis (169). Attrition and inconsistencies in participant counts, dosing schedules, and follow-up descriptions limit confidence in the findings. This evidence supports further product-specific clinical investigation rather than established efficacy across diabetes and its complications.

## Challenges and perspectives

Despite the remarkable progress in understanding the immunomodulatory and therapeutic potential of MSC-EVs in diabetes, several challenges impede their translation from bench to bedside. One of the primary hurdles lies in the large-scale production of MSC-EVs with consistent quality. Current isolation techniques, such as ultracentrifugation, density gradient centrifugation, and SEC, are time-consuming, labor-intensive, and often result in low yields. For instance, ultracentrifugation, a widely used method, requires high-speed centrifugation for extended periods, which may damage the EVs and lead to variable purity. Additionally, the lack of standardized protocols for MSC culture and EV isolation across different laboratories makes it difficult to compare results from various studies.

The dosing regimens in **Supplementary Table S1** should be interpreted in relation to the EV preparation, animal model, administration route, and treatment timing. Protein-based and particle-based doses cannot be directly equated across preparations. These data inform the design of product-specific dose-response and time-course studies rather than define a common effective dose for clinical use (23).

A significant concern revolves around the precise targeting and delivery of MSC-EVs to the desired tissues within the diabetic body. While MSC-EVs possess a certain degree of homing ability, enhancing their specificity to pancreatic islets, damaged blood vessels in DR or nephropathy, and inflamed tissues in DFUs, remains challenging. Developing strategies to functionalize MSC-EVs with targeting ligands, such as peptides or antibodies that recognize specific cell surface markers in diabetic tissues, could potentially improve their delivery efficiency. However, this approach necessitates careful consideration of potential immunogenicity and off-target effects.

Additionally, the long-term safety of MSC-EVs in humans has yet to be fully clarified. Although preclinical studies have demonstrated promising outcomes with minimal adverse effects, the absence of large-scale, long-term clinical trials complicates the prediction of rare or delayed adverse events. Concerns persist regarding the potential for EV-mediated gene transfer to unintended cells, which could result in unforeseen genetic alterations and risks such as tumorigenesis. Moreover, the effects of repeated administrations of MSC-EVs on the immune system over time are not well understood.

Looking to the future, technological advancements hold great promise for overcoming these challenges. The development of microfluidic and nanofluidic platforms may enable more efficient and precise isolation of MSC-EVs, while also reducing the cost and time required for production. These platforms can precisely control the microenvironment for EV isolation, leading to higher yields and better-defined products. In terms of delivery, the use of biomimetic nanoparticles or engineered carriers that mimic the properties of natural EVs could improve the targeting and delivery of MSC-EVs. For example, exosome-mimicking nanoparticles can be designed to encapsulate therapeutic cargo and display targeting molecules on their surface.

Moreover, future research should focus on personalized medicine approaches utilizing MSC-EVs. Given the heterogeneity of diabetes in terms of etiology, pathophysiology, and patient response, tailoring MSC-EV-based therapies to individual patients could optimize treatment outcomes. This may involve stratifying patients based on genetic profiles, disease stage, and immune status to determine the most appropriate MSC-EV dosage, source, and delivery method. Additionally, combination therapies that integrate MSC-EVs with existing diabetes treatments, such as metformin, insulin, or GLP-1 analogs, could potentially enhance therapeutic efficacy and provide more comprehensive management of diabetes and its complications.

## Conclusion

MSC-EVs have emerged as a versatile and promising therapeutic modality in the field of diabetes research, with their significance extending beyond mere biological carriers to encompass a sophisticated regulatory network that integrates immune modulation, metabolic balance, and tissue regeneration. Their inherent ability to traverse biological barriers and interact with diverse target cells allows them to address the multi-systemic nature of diabetes, from pancreatic β-cell protection to the mitigation of microvascular and neurological complications. The cumulative evidence from mechanistic studies underscores their capacity to harmonize disrupted signaling pathways, reset immune homeostasis, and reactivate endogenous repair mechanisms, thereby offering a holistic approach that transcends the limitations of conventional symptom-based therapies.

The journey from preclinical discovery to clinical application of MSC-EVs necessitates a synergistic advancement in technological refinement and conceptual innovation. Standardization of production processes, including isolation, purification, and characterization, remains a cornerstone for ensuring reproducibility and safety, while functional engineering strategies hold the key to enhancing targeting efficiency and therapeutic potency. Future endeavors should focus on unraveling the long-term biological fate of MSC-EVs *in vivo* and their interplay with the host’s dynamic physiological milieu, which will be critical for tailoring personalized therapeutic regimens. As research continues to bridge the gap between bench and bedside, MSC-EVs are poised to redefine the landscape of diabetes management, offering new avenues for disease modification and complication prevention.

## Glossary

3D hUCB-MSC-EVs: Three-dimensional-cultured human umbilical cord blood MSC-EVs
ADSC-EVs: Adipose-derived mesenchymal stem cell EVs
AGEs: Advanced glycation end products
AMPK/mTOR: adenosine monophosphate-activated protein kinase/mammalian target of rapamycin
ApoE−/−: apolipoprotein E-deficient
APRIL: a proliferation-inducing ligand
Arg-1: Arginase-1
BAD: BCL-2-associated death promoter
BAFF: B-cell-activating factor
BAX: BCL-2-associated X protein
Bcl-2: B-cell lymphoma 2
BMD: Bone mineral density
BMSC-EVs: Bone marrow mesenchymal stem cell-derived EVs
BRB: Blood-retinal barrier
Ca2+: calcium ion
ccSMCs: Corpus cavernosum smooth muscle cells
CD9: Cluster of differentiation 9
cMSC-EVs: Clonal MSC-Evs
CRP: C-reactive protein
CSEVs: Calcium silicate-stimulated adipose-derived MSC-EVs
DAPK1: Death-associated protein kinase 1
DCs: Dendritic cells
DFUs: Diabetic foot ulcers
DKD: Diabetic kidney disease
DMED: Diabetes mellitus-induced erectile dysfunction
DOP: Diabetic osteoporosis
DPN: Diabetic peripheral neuropathy
DR: Diabetic retinopathy
ESC-NVs: Embryonic stem cell-derived EV-mimetic nanovesicles
ESRD: End-stage renal disease
EXE-EVs: Exercise-induced circulating EVs
FGF: Fibroblast growth factor
FOXO1: Forkhead box O1
FOXP3+: Forkhead box P3-positive
GAD65: glutamic acid decarboxylase 65
GLP-1: Glucagon-like peptide-1
GLUT4: glucose transporter 4
GMNPs: Gold magnetic nanoparticles
HA: Hyaluronic acid
HAL: hexyl 5-aminolevulinate hydrochloride
HDAC4: Histone Deacetylase 4
HFD/STZ: high-fat diet/streptozotocin
Hhip: Hedgehog interacting protein
HIF-1α: Hypoxia-inducible factor 1 alpha
HLA: Human leukocyte antigen
HO-1: Heme oxygenase-1
HSPs: Heat shock proteins
HOTAIR HOX: transcript antisense RNA
IL-6: Interleukin-6
IDO: indoleamine 2,3-dioxygenase
IFN-γ: Interferon-γ
IGF: Insulin-like growth factor
ILC2s: Innate lymphoid cells are functionally heterogeneous: group 2 innate lymphoid cells
iNOS: Inducible nitric oxide synthase
IRAK1: Interleukin-1 receptor-associated kinase 1
IRS1: Insulin receptor substrate 1
LDL: Low-density lipoprotein
lncRNAs: Long non-coding RNAs
M2-EVs: M2 macrophage-derived Evs
MAPK: mitogen-activated protein kinase
MDVs: Mitochondrial-derived vesicles
MHC: major histocompatibility complex
miRNAs: MicroRNAs
MISEV2023: Minimal Information for Studies of Extracellular Vesicles 2023
MMP14: Matrix metalloproteinase 14
mRNAs: Messenger RNAs
MSC-EVs: Mesenchymal stem cell-derived extracellular vesicles
MSC-sEVs: mesenchymal stem cell-derived small extracellular vesicles
MVBs: Multivesicular bodies
NET: neutrophil extracellular trap
NF-κB: Nuclear factor kappa-light-chain-enhancer of activated B cells
NK: natural killer
NLRP3: NOD-like receptor pyrin domain-containing protein 3
NO: Nitric oxide
NRF2: Nuclear factor erythroid 2-related factor 2
PBMCs: Peripheral blood mononuclear cells
PDK4: Pyruvate dehydrogenase kinase 4
PD-L1: Programmed death-ligand 1
PDX1: pancreatic and duodenal homeobox 1
PEG: Polyethylene glycol
PI3K/Akt: Phosphoinositide 3-kinase/protein kinase B
PINK1: PTEN-induced kinase 1
PLCG1: Phospholipase C gamma 1
Plxnb1: Plexin B1
PKC: Protein kinase C
PP2A: Protein phosphatase 2A
PTEN: Phosphatase and tensin homolog
PTP1B: Protein tyrosine phosphatase 1B
PVT1: Plasmacytoma variant translocation 1
RAB27B: Ras-related protein Rab-27B
RANKL: Receptor activator of nuclear factor-κB ligand
ROS: Reactive oxygen species
SASP: Senescence-associated secretory phenotype
SEC: Size-exclusion chromatography
SIRT1: Sirtuin 1
SMD: standardized-mean- difference
SNX9: Sorting nexin 9
T1D: Type 1 diabetes
T2D: Type 2 diabetes
TcPO_2_: Transcutaneous partial pressure of oxygen
TGF-β: Transforming growth factor-β
TLR4: Toll-like receptor 4
TNF-α: Tumor necrosis factor-α
TRAF6: Tumor necrosis factor receptor-associated factor 6
Tregs: Regulatory T cells
USC-EVs: Urine-derived stem cell EVs
VEGF: Vascular endothelial growth factor
WHO ICTRP: World Health Organization International Clinical Trials Registry Platform
Wnt/β-catenin: Wingless-type MMTV integration site family/β-catenin signaling
