# Supplementary material for: Mesenchymal stem cell-derived extracellular vesicles in diabetes: immunomodulation and therapeutic prospects
Source: Front Immunol. 2026 Oct 19;17:1918124. doi: 10.3389/fimmu.2026.1918124 (PMC13725451; doi:10.3389/fimmu.2026.1918124)
Supplement: Supplementary file 1 [file Table1.docx]

**Table S1. Preclinical animal studies of MSC-EVs in diabetes and its complications: sources, doses, routes, and treatment schedules.**

| **Study** | **MSC source and EV preparation** | **Animal model** | **EV dose per administration** | **Route** | **Treatment initiation** | **Dosing schedule** | **Follow-up / assessment** |
| --- | --- | --- | --- | --- | --- | --- | --- |
| Shigemoto-Kuroda et al. 2017 (1) | Human BM-MSC-derived EVs; diabetes arm only | NOD/scid mice receiving splenocytes from diabetic NOD mice | 3 or 30 μg EV protein/injection | IV | Immediately after splenocyte transfer (day 0) | Days 0 and 4; two injections | Glycemia monitored; EV-arm tissue collection on day 58 |
| Ou et al. 2024 (2) | Human umbilical cord blood MSC-EVs | Female NOD/Ltf mice; early autoimmune diabetes model | 2 mg/kg EV protein in 200 μL PBS | Tail-vein IV | At 4 weeks of age | Twice weekly for 8 weeks | After 8 weeks of treatment |
| Yap et al. 2022 (3) | Human UC-MSC sEVs | Male SD rats; HFD/STZ-induced T2D | 0.25, 0.5 or 1 mg/kg; separate dose groups | Tail-vein IV | After diabetes confirmation, approximately study week 8 | Every 3 days; 5 doses | 15-day intervention period |
| Cui et al. 2024 (4) | Human Wharton’s jelly MSC-sEVs; apelin-enriched and control preparations | C57BL/6J mice; HFD/STZ-induced T2D | 7 × 10¹¹ particles per mouse | Tail-vein IV | After T2D model confirmation; exact interval NS | Repeat-dosing frequency NS | Glucose/insulin tolerance testing 5 weeks after treatment |
| Siriarchavatana et al. 2025(5) | 3D-cultured canine adipose MSC-sEVs | Male C57BL/6NJ mice; STZ-induced insulin-deficient diabetes | 1 × 10⁷ particles/kg; 0.1 mL suspension | IV | 10 days after the final STZ dose (day 0) | Compared 1 dose (day 0), 2 doses (days 0, 7), and 3 doses (days 0, 7, 14) | Day 21 after first treatment |
| Shi et al. 2017 (6) | Human gingival MSC EVs in chitosan/silk hydrogel sponge | Male SD rats; diet/STZ diabetes; 10-mm dorsal full-thickness wound | 150 μg/100 μL PBS loaded onto a 13 × 13-mm dressing | Topical | At wound creation | Dressing replaced every 3 days | 1 and 2 weeks after wounding |
| Yu et al. 2020 (7) | Human BM-MSCs; unprimed or atorvastatin-primed EVs | Male SD rats; STZ diabetes; 2-cm dorsal wound | NS for the animal wound experiment | Periwound SC, ≥6 sites | At wound creation after diabetes confirmation | Initial local administration; repeat schedule NS | Days 0, 3, 7 and 14; histology days 7 and 14 |
| Liu et al. 2020 (8) | Human BM-MSCs; unprimed or melatonin-primed EVs | Male SD rats; STZ diabetes; 2-cm dorsal full-thickness wound | NS for the animal wound experiment | Periwound SC, ≥6 sites | At wound creation after diabetes confirmation | Initial local administration; repeat schedule NS | Days 0, 3, 7 and 14; histology days 7 and 14 |
| Wang et al. 2021 (9) | Human adipose MSC-EVs; normoxic or hypoxia-preconditioned | BALB/c nude mice; HFD/STZ diabetes and 0.8 × 0.8 cm dorsal wound | 2 mg EV protein in 100 μL PBS | Periwound SC, 4 sites | Immediately after wounding; established diabetes | Initial local treatment; repeat schedule NS | Wound closure on days 0, 3, 7, 14 |
| Hu et al. 2021 (10) | Rat BM-MSCs; unprimed or pioglitazone-primed EVs | Male SD rats; STZ diabetes; 15-mm full-thickness skin wound | 100 μg in 100 μL PBS | Periwound SC, ≥6 sites | At wound creation after diabetes confirmation | Initial local injection; repeat regimen NS | Days 0, 3, 7, 10 and 14; tissue collection day 14 |
| Wu et al. 2023 (11) | Rat adipose MSC-EVs, with or without IRF1 overexpression; EV injection arms only | Male SD rats; STZ-induced diabetes, 5-mm dorsal foot wound | 0.5 mL at 1 × 10¹¹ particles/mL | Local injection into wound edge and centre | After wound creation, following 2-week diabetes confirmation | Once daily for 2 days | Wound assessment through day 14 |
| Yang et al. 2023 (12) | Human UC-MSC EVs, free or in chitosan hydrogel | SD rats; diet/STZ diabetes; 10-mm dorsal full-thickness wound | 1 × 10¹⁰ particles/wound | Topical | At wound creation | Initial application; dressing changes on days 5, 10 and 15; EV re-dosing NS | Days 5, 10 and 15 |
| Lu et al. 2024 (13) | Human UC-MSC EVs; mitochondrial-function controls | STZ-diabetic mice; WT/Padi4−/− mechanistic groups; 5-mm dorsal wounds | Approximately 7 × 10¹⁰ particles/100 μL PBS | Systemic; exact route NS | NS | Frequency and duration NS | Days 0, 3, 7 and 14 after wounding |
| Wang et al. 2024 (14) | Human umbilical cord MSCs; control or CHIR99021-primed EVs | BALB/c mice; STZ diabetes; 8-mm dorsal wound | 200 μg in 200 μL PBS | Local injection at four periwound sites | At wound creation after 4 weeks of diabetes stabilization | Once every 7 days | Days 0, 3, 7, 10 and 14 |
| Lin et al. 2025 (15) | Human adipose MSC EVs; calcium-silicate priming; collagen hydrogel | Male New Zealand White rabbits; alloxan diabetes; 1.5-cm-square dorsal wounds | EV amount per wound NS | Topical hydrogel placement | At wound creation | Initial placement; repeat frequency NS | Days 14 and 21 |
| Zhao et al. 2025 (16) | Human UC-MSC EVs; endothelial-targeting aptamer; HA carrier | Female C57BL/6 mice; STZ diabetes; 1-cm dorsal full-thickness wounds | 150 μg EV preparation per diabetic mouse | Local wound delivery in HA | After wound creation; exact interval NS | Repeat schedule NS | Days 7 and 14 after treatment |
| Reddy et al. 2023 (17) | Human BM-MSC sEVs loaded with bevacizumab | Wistar rats; STZ-induced diabetic retinopathy | 50 μg sEV protein/10 μL; bevacizumab 60–74 μg/mL | Intravitreal | 4 months after STZ | Single EV–drug injection | 1, 2 and 3 months after injection |
| Fan et al. 2026 (18) | Mouse BM-MSC EVs; IL-4-loaded and microglia-targeted preparations; STZ arm only | C57BL/6 mice; STZ-induced diabetic retinopathy | 1 μg EV preparation in 1 μL | Intravitreal | After diabetes confirmation; exact initial interval NS | Monthly | At 10 weeks of diabetes |
| Nagaishi et al. 2016 (19) | Rat BM-MSC-derived EVs; isolated-EV arm only | STZ-induced diabetic rats; renal injury | 5.3 × 10⁷ EVs (CD9-ELISA estimate) in 200 μL PBS/kidney | Unilateral renal subcapsular injection | After STZ-induced diabetes; exact interval NS | One injection | 7 and 14 days after injection |
| Grange et al. 2019 (20) | Human BM-MSC EVs; MSC arm only | Male NSG mice; STZ-induced diabetic nephropathy | 1 × 10¹⁰ particles | IV | Day 30 after diabetes onset | Weekly over 4 weeks; 5 injections reported | Day 60 after diabetes onset |
| Ren et al. 2023 (21) | Adipose MSC-EVs; donor species NS in the examined Methods | Male C57BL/KsJ db/db mice; diabetic nephropathy | 100 μg/mL reported; injection volume and total EV dose NS | Tail-vein IV | After nephropathy confirmation at 12 weeks of age | Repeat frequency NS | 12-week experimental period |
| Li et al. 2025 (22) | Human placental MSC EVs; miR-99b-5p mechanistic perturbation | Male C57BL/6 mice; STZ-induced diabetic kidney disease | 100 μg/200 μL PBS | Tail-vein IV | 8 weeks after the last STZ injection | Weekly for 12 consecutive weeks | 20 weeks after the last STZ injection |
| Song et al. 2026 (23) | Mouse BM-MSC EVs; negative-control or miR-143-3p-inhibitor preparations | Male C57BL/6J mice; HFD/STZ-induced diabetic kidney disease | 100 μg EV preparation in 200 μL 0.9% NaCl | Tail-vein IV | After 8 additional HFD weeks following diabetes confirmation; UACR >30 mg/g | Weekly for 4 weeks | After 4 weeks of EV treatment |
| Wang et al. 2026 (24) | Human adipose MSC-EVs; unmodified and miR-21-enriched arms | Male C57BL/KsJ db/db mice receiving HFD; diabetic nephropathy | 100 μg in 0.2 mL PBS | Tail-vein IV | Experimental week 3 | Unmodified EVs: 3 times in week 1, then twice weekly for 3 weeks; miR-21 EVs: 3 times weekly for 3 weeks | After the respective 4- or 3-week regimens |
| Liu et al. 2025 (25) | Mouse BM-MSC EVs | Male ApoE−/− mice; HFD/STZ diabetes-associated atherosclerosis | 100 μg/200 μL | Tail-vein IV | After diabetes confirmation; approximately study week 7 | Twice weekly for 12 weeks | Study week 19 |
| Wang et al. 2022 (26) | Rat BM-MSC EVs; normal, diabetic or miR-140-3p-enriched preparations | Male SD rats; diet/STZ diabetes; 3-mm femoral metaphyseal defect | 240 μg EV preparation in Matrigel | Local, into bone defect | At defect surgery | Single placement | 2 and 8 weeks after surgery |
| Xu et al. 2023 (27) | Rat BM-MSC EVs; anti-CD63 gold/magnetic nanoparticle targeting; miR-150-5p manipulation | Male SD rats; STZ-induced diabetic osteoporosis | EV suspension: 100 μL; EV mass/particle count NS; GMNPE: 10 mg/kg, separately reported | Tail-vein IV plus external femoral magnetic field | After 8-week model establishment | Injection described; repeat frequency NS | 8 weeks after treatment |
| Xu et al. 2024 (28) | Rat BM-MSC EVs coupled to magnetic nanoparticles; miR-15b-5p studies | STZ-diabetic rats with osteoporosis; strain inconsistently reported | 400 μL nanoparticle suspension/kg reported; EV mass/particle count NS | Tail-vein IV; external femoral magnetic field | After diabetic model establishment; exact interval NS | Repeat frequency NS | Post-EV follow-up interval NS |
| Fan et al. 2020 (29) | Mouse MSC-derived EVs | Male db/db mice; established diabetic peripheral neuropathy | 1 × 10⁹ particles/animal/injection | Tail-vein IV | 20 weeks of age | Weekly for 8 consecutive weeks | Every 2 weeks; tissue collection at week 8 |
| Fan et al. 2021 (30) | Mouse BM-MSC EVs; naïve or miR-146a-enriched | Male db/db mice; diabetic peripheral neuropathy | 1 × 10⁹ particles/injection | Tail-vein IV | 20 weeks of age | Weekly for 4 weeks | 2 and 4 weeks after treatment initiation |

Abbreviations: MSC, mesenchymal stem/stromal cell; EV, extracellular vesicle; sEV, small EV; BM, bone marrow; UC, umbilical cord; HFD, high-fat diet; STZ, streptozotocin; SD, Sprague–Dawley; IV, intravenous; SC, subcutaneous; PBS, phosphate-buffered saline; HA, hyaluronic acid; T2D, type 2 diabetes; UACR, urinary albumin-to-creatinine ratio; WT, wild type.

# References

1.	Shigemoto-Kuroda T, Oh JY, Kim D-k, Jeong HJ, Park SY, Lee HJ, et al. MSC-derived Extracellular Vesicles Attenuate Immune Responses in Two Autoimmune Murine Models: Type 1 Diabetes and Uveoretinitis. Stem cell reports. 2017;8(5):1214-25.

2.	Ou Y, Yang Y, Wang Y, Su H, Zhou Y-k, Wruck W. Effects of Extracellular Vesicles Derived from Human Umbilical Cord Blood Mesenchymal Stem Cells on Cell Immunity in Nonobese Mice. Stem Cells International. 2024;2024(1).

3.	Yap SK, Tan KL, Abd Rahaman NY, Saulol Hamid NF, Ooi DJ, Tor YS, et al. Human Umbilical Cord Mesenchymal Stem Cell-Derived Small Extracellular Vesicles Ameliorated Insulin Resistance in Type 2 Diabetes Mellitus Rats. Pharmaceutics. 2022;14(3):649.

4.	Cui J, Wang M, Zhang W, Sun J, Zhang Y, Zhao L, et al. Enhancing insulin sensitivity in type 2 diabetes mellitus using apelin-loaded small extracellular vesicles from Wharton’s jelly-derived mesenchymal stem cells: a novel therapeutic approach. Diabetology & Metabolic Syndrome. 2024;16(1).

5.	Siriarchavatana P, Oontawee S, Rodprasert W, Thongsit A, Somparn P, Na Nan D, et al. Small extracellular vesicles produced from canine adipose-derived mesenchymal stem cells mitigate diabetes mellitus in streptozotocin-induced mice via β-cell revitalization. BMC Veterinary Research. 2025;21(1).

6.	Shi Q, Qian Z, Liu D, Sun J, Wang X, Liu H, et al. GMSC-Derived Exosomes Combined with a Chitosan/Silk Hydrogel Sponge Accelerates Wound Healing in a Diabetic Rat Skin Defect Model. Frontiers in Physiology. 2017;8.

7.	Yu M, Liu W, Li J, Lu J, Lu H, Jia W, et al. Exosomes derived from atorvastatin-pretreated MSC accelerate diabetic wound repair by enhancing angiogenesis via AKT/eNOS pathway. Stem Cell Research & Therapy. 2020;11(1).

8.	Liu W, Yu M, Xie D, Wang L, Ye C, Zhu Q, et al. Melatonin-stimulated MSC-derived exosomes improve diabetic wound healing through regulating macrophage M1 and M2 polarization by targeting the PTEN/AKT pathway. Stem cell research & therapy. 2020;11(1):1-15.

9.	Wang J, Wu H, Peng Y, Zhao Y, Qin Y, Zhang Y, et al. Hypoxia adipose stem cell-derived exosomes promote high-quality healing of diabetic wound involves activation of PI3K/Akt pathways. Journal of Nanobiotechnology. 2021;19(1).

10.	Hu Y, Tao R, Chen L, Xiong Y, Xue H, Hu L, et al. Exosomes derived from pioglitazone-pretreated MSCs accelerate diabetic wound healing through enhancing angiogenesis. Journal of Nanobiotechnology. 2021;19(1).

11.	Wu M, Tu J, Huang J, Wen H, Zeng Y, Lu Y. Exosomal IRF1-loaded rat adipose-derived stem cell sheet contributes to wound healing in the diabetic foot ulcers. Molecular Medicine. 2023;29(1).

12.	Yang S, Chen S, Zhang C, Han J, Lin C, Zhao X, et al. Enhanced therapeutic effects of mesenchymal stem cell-derived extracellular vesicles within chitosan hydrogel in the treatment of diabetic foot ulcers. Journal of materials science Materials in medicine. 2023;34(9):43-14.

13.	Lu W, Li X, Wang Z, Zhao C, Li Q, Zhang L, et al. Mesenchymal stem cell-derived extracellular vesicles accelerate diabetic wound healing by inhibiting NET-induced ferroptosis of endothelial cells. International journal of biological sciences. 2024;20(9):3515-29.

14.	Wang L, Chen J, Song J, Xiang Y, Yang M, Xia L, et al. Activation of the Wnt/β-catenin signalling pathway enhances exosome production by hucMSCs and improves their capability to promote diabetic wound healing. Journal of Nanobiotechnology. 2024;22(1).

15.	Lin Y-H, Chen Y, Liu E-W, Chen M-C, Yu M-H, Chen C-Y, et al. Immunomodulation effects of collagen hydrogel encapsulating extracellular vesicles derived from calcium silicate stimulated-adipose mesenchymal stem cells for diabetic healing. Journal of Nanobiotechnology. 2025;23(1).

16.	Zhao N, Guo Z-Z, Bai S-F, Gao Y-F, Lei J, Li P, et al. Endothelial cell-targeting aptamer-empowered exosomes accelerate wound healing by promoting specialized angiogenesis in type 1 diabetic mice. Stem Cell Research & Therapy. 2025;17(1).

17.	Reddy SK, Ballal AR, Shailaja S, Seetharam RN, Raghu CH, Sankhe R, et al. Small extracellular vesicle-loaded bevacizumab reduces the frequency of intravitreal injection required for diabetic retinopathy. Theranostics. 2023;13(7):2241-55.

18.	Fan Y, Ge P, Wang X, Xu J, Wang J, Li H, et al. Microglia-specific interleukin-4 delivery by engineered extracellular vesicles restores inner blood-retinal barrier in diabetic retinopathy via GAS6-MERTK pathway. Journal of Nanobiotechnology. 2025;24(1).

19.	Nagaishi K, Mizue Y, Chikenji T, Otani M, Nakano M, Konari N, et al. Mesenchymal stem cell therapy ameliorates diabetic nephropathy via the paracrine effect of renal trophic factors including exosomes. Scientific Reports. 2016;6(1).

20.	Grange C, Tritta S, Tapparo M, Cedrino M, Tetta C, Camussi G, et al. Stem cell-derived extracellular vesicles inhibit and revert fibrosis progression in a mouse model of diabetic nephropathy. Scientific reports. 2019;9(1):4468.

21.	Ren P, Qian F, Fu L, He W, He Q, Jin J, et al. Adipose-derived stem cell exosomes regulate Nrf2/Keap1 in diabetic nephropathy by targeting FAM129B. Diabetology and metabolic syndrome. 2023;15(1):149-10.

22.	Li R, Tao H, Pan K, Li R, Guo Z, Chen X, et al. Extracellular vesicles derived from mesenchymal stem cells alleviate renal fibrosis via the miR-99b-5p/mTOR/autophagy axis in diabetic kidney disease. Stem cell research & therapy. 2025;16(1):142-14.

23.	Song W, Wang J, Guan L, Zou Y, Liu J, Chen W, et al. Mesenchymal Stem Cell-Derived Exosomes miR-143-3p Attenuates Diabetic Kidney Disease by Enhancing Podocyte Autophagy via Bcl-2/Beclin1 Pathway. Biomedicines. 2026;14(1):184.

24.	Wang Y, Lu Y, Zhao Y, Xu J, Gan L, Yang C, et al. Dual role of miR-21-enriched MSC-derived exosomes in diabetic nephropathy: therapeutic potential versus p53-driven pathogenicity. BMC Nephrology. 2026;27(1).

25.	Liu L, An Z, Zhang H, Wan X, Zhao X, Yang X, et al. Bone marrow mesenchymal stem cell-derived extracellular vesicles alleviate diabetes-exacerbated atherosclerosis via AMPK/mTOR pathway-mediated autophagy-related macrophage polarization. Cardiovascular diabetology. 2025;24(1):48-16.

26.	Wang N, Liu X, Tang Z, Wei X, Dong H, Liu Y, et al. Increased BMSC exosomal miR-140-3p alleviates bone degradation and promotes bone restoration by targeting Plxnb1 in diabetic rats. Journal of nanobiotechnology. 2022;20(1):97-20.

27.	Xu C, Wang Z, Liu Y, Wei B, Liu X, Duan K, et al. Extracellular vesicles derived from bone marrow mesenchymal stem cells loaded on magnetic nanoparticles delay the progression of diabetic osteoporosis via delivery of miR-150-5p. Cell biology and toxicology. 2023;39(4):1257-74.

28.	Xu C, Wang Z, Liu Y, Duan K, Guan J. Delivery of miR-15b-5p via magnetic nanoparticle-enhanced bone marrow mesenchymal stem cell-derived extracellular vesicles mitigates diabetic osteoporosis by targeting GFAP. Cell biology and toxicology. 2024;40(1):52.

29.	Fan B, Li C, Szalad A, Wang L, Pan W, Zhang R, et al. Mesenchymal stromal cell-derived exosomes ameliorate peripheral neuropathy in a mouse model of diabetes. Diabetologia. 2020;63(2):431-43.

30.	Fan B, Chopp M, Zhang ZG, Liu XS. Treatment of diabetic peripheral neuropathy with engineered mesenchymal stromal cell-derived exosomes enriched with microRNA-146a provide amplified therapeutic efficacy. Experimental neurology. 2021;341:113694-.
