# Supplementary material for: Mesenchymal stem cell-derived extracellular vesicles in diabetes: immunomodulation and therapeutic prospects
Source: Front Immunol. 2026 Oct 19;17:1918124. doi: 10.3389/fimmu.2026.1918124 (PMC13725451; doi:10.3389/fimmu.2026.1918124)
Supplement: Supplementary file 2 [file Table2.docx]

**Table 1. Comparison of Mesenchymal Stem Cells and Their Extracellular Vesicles in the Treatment of Diabetes**

| **Comparison Dimension** | **Mesenchymal Stem Cells(MSCs)** | **MSC-Derived Extracellular Vesicles (MSC-EVs)** |
| --- | --- | --- |
| **Cell Source** | Derived from bone marrow, adipose tissue, umbilical cord, and other tissues. Functional properties vary with tissue source and culture conditions. | Released by MSCs from the corresponding tissue source and containing selected donor-cell-derived components. Their composition and biological activity depend on the donor cells and culture conditions. |
| **Mechanism** | 1.Human bone marrow-, adipose tissue-, and umbilical cord Wharton’s jelly-derived MSCs have been induced into insulin-producing cells under defined in vitro conditions(11-13); 2. Paracrine mediators include TGF-β1 and VEGF, with secretion profiles varying among bone marrow-, adipose tissue-, and Wharton’s jelly-derived MSCs (14, 15); 3. Interactions with immune cells can suppress T-cell proliferation and modulate macrophage phenotype(16, 17). | 1. EV cargo includes miRNAs, proteins, and lipids. Examples include miR-146a in engineered bone marrow MSC-EVs, miR-26a-5p in adipose MSC-EVs, and PD-L1 or SIRT1 in selected engineered preparations, 2. TLR4/NF-κB suppression and macrophage phenotype modulation have been reported in specific experimental models; 3. Akt-associated insulin signaling and Wnt/β-catenin-associated bone repair have been reported in distinct diabetic models. |
| **Immunogenicity** | Not intrinsically immune privileged; allogeneic immune rejection can occur. | MHC class I has been detected in some preparations, and MSC-EVs can activate innate immune signaling. Their cell-free nature does not establish absence of immunogenicity; immune responses require preparation-specific assessment. |
| **Safety** | An updated meta-analysis of intravascular MSC trials found increased fever but no detected increase in malignancy. Safety conclusions remain limited to the products, populations, and follow-up evaluated | Cell-free and unable to replicate independently, but not inherently risk-free. Long-term safety and potential adverse effects require preparation-specific evaluation. |
| **Tissue Targeting** | Intravenous administration can result in pulmonary first-pass retention, limiting delivery to downstream tissues. | Biodistribution depends on cell source and administration route. Engineering or carrier-assisted strategies can improve targeted delivery in specific experimental settings. |
| **Production and Storage** | Requires controlled cell expansion and validated cryopreservation procedures, with assessment of post-thaw viability and functional activity. | Scalable production has been demonstrated for selected MSC-EV products. Separation quality and storage stability depend on the methods, formulation, and storage conditions. |
| **Durability of Efficacy** | Cell persistence can be brief after intravenous administration in animal models; persistence alone should not be equated with the duration of therapeutic benefit. | Repeated dosing has been used in diabetic neuropathy studies, and hydrogel delivery can sustain local EV release. These findings do not establish a universal duration of therapeutic benefit. |
| **Effect on Diabetic Complications** | Tissue-repair effects have been investigated in diabetic wound models(18). Bone marrow MSC therapy has also been evaluated in patients with diabetic critical limb ischemia and foot ulcers. | Reduced renal fibrosis and improved neuropathy outcomes have been reported in diabetic animal models using specific MSC-EV preparations. These findings are preclinical and do not establish efficacy across all diabetic complications. |
| **Clinical Translation Potential** | Evaluated in clinical studies of T1D and diabetic ischemic foot disease. Findings remain specific to the cell product, patient population, and trial design. | A cell-free therapeutic approach supported by preclinical studies. Clinical translation requires standardized manufacturing, quality control, and product-specific evaluation of safety and efficacy. |

BMSCs stands for Bone Marrow Mesenchymal Stem Cells; ADSCs stands for Adipose-Derived Stem Cells; hUCMSC-EVs stands for Human Umbilical Cord Mesenchymal Stem Cell-Derived Extracellular Vesicles; miR stands for microRNA; PD-L1 stands for Programmed Death-Ligand 1; SIRT1 stands for Sirtuin 1; TLR4 stands for Toll-Like Receptor 4; NF-κB stands for Nuclear Factor-Kappa B; PI3K stands for Phosphatidylinositol 3-Kinase; Akt stands for Protein Kinase B; TGF-β stands for Transforming Growth Factor-Beta; VEGF stands for Vascular Endothelial Growth Factor; MHC stands for Major Histocompatibility Complex. MSC-EVs is used here as an umbrella term and does not imply that all preparations are exosomes or have the same size distribution. This table summarizes findings from different studies and does not establish that MSCs or MSC-EVs are uniformly superior in efficacy, safety, targeting, or durability. General methodological studies are used for context rather than as direct evidence of efficacy in diabetes.
