# Supplementary material for: Mesenchymal stem cell-derived extracellular vesicles in diabetes: immunomodulation and therapeutic prospects
Source: Front Immunol. 2026 Oct 19;17:1918124. doi: 10.3389/fimmu.2026.1918124 (PMC13725451; doi:10.3389/fimmu.2026.1918124)
Supplement: Supplementary file 3 [file Table3.docx]

**Table 2. Registered clinical studies of MSC-EVs in diabetes and its complications.**

| **Trial ID / condition** | **Phase / stage** | **Sponsor** | **Intervention / route** | **Endpoints** | **Status / record date** | **Available results** |
| --- | --- | --- | --- | --- | --- | --- |
| NCT02138331 [1] T1D | 2/3 in phase field | General Committee of Teaching Hospitals and Institutes, Egypt | Cord-blood MSC-EV fractions described as exosomes and microvesicles; IV administrations 7 days apart. | Primary: total daily insulin dose at 3 months. Other listed measures: β-cell mass and HbA1c. | Unknown; last known: enrolling by invitation. Record: 14 May 2014. | No posted results in the retrieved record; no trial-specific clinical results publication identified. |
| NCT07693036 [2] T2D (HOPE-T2DM) | 1/2 (b) | Peking University First Hospital, China; collaborator: ECHO Biotech | hUC-MSC-EVs (GoldenExo/NATX); IV weekly for 8 doses. Dose escalation, then randomized EV-free-buffer-controlled stage. | Primary: HbA1c change at week 8. Selected secondary: CGM time in range, insulin/C-peptide and longer-term metabolic measures. Safety monitored. | Recruiting. Record: 9 July 2026. | No results posted in the retrieved record; no trial-specific clinical results publication identified. |
| NCT06812637 [3,7] DFU | 1 (b) | Kafrelsheikh University, Egypt | Topical WJ-MSC-EV gel plus SOC; comparators: SOC alone and vehicle gel plus SOC. Administration frequency differs within the publication (c). | Publication: ulcer-area reduction. | Completed in registry-derived copy (b). Record date in copy: 29 April 2025. | 2025 report: 110 randomized, 85 analyzed; mean healing time reported as 6 vs 20 weeks. Attrition and internal reporting discrepancies limit interpretation (c). |
| ChiCTR2500103555 [4] T2D with DFU | Exploratory / pilot study (asregistered) | Chinese PLA General Hospital, China | Local subcutaneous hUC-MSC exosome administration versus conventional treatment; multicenter randomized study. | Wound healing rate, overall response measure and positive sweating-test rate. Endpoint time points not specified in the retrieved record. | Not yet recruiting in cited version. Version 1.0: 30 May 2025. | No outcome results identified in the retrieved registry information or trial-ID-linked publications. |
| ChiCTR2600121779 [5] T2D with DFU | Exploratory / pilot study (as registered) | The Second Affiliated Hospital of Zhejiang University School of Medicine, China | Topical MSC exosome suspension after debridement, then hydrogel cover; 5×10¹⁰, 1×10¹¹ or 2×10¹¹ particles. Tissue source not specified. | Primary: 28-day DLT, treatment-related AEs/SAEs, MTD/RP2D. Secondary: 12-week closure, 4-week area reduction, healing time and amputation. | Not yet recruiting in cited record. Updated: 2 April 2026. | No outcome results identified in the retrieved registry information or trial-ID-linked publications. |
| NCT07480161 [6] Diabetic ED | 2/3 (b) | Ankara City Hospital Bilkent, Türkiye | Separate arms: single intracavernosal UC-MSC exosomes (75 μg), live UC-MSCs (5×10⁶ cells), or saline. Not combined cell–EV therapy. | Primary: IIEF-5 and EHS at months 1, 3, 6 and 12. Secondary: penile Doppler measures and adverse events. | Recruiting. Record: 18 March 2026. | No results posted in the retrieved record; no trial-specific clinical results publication identified. |

Abbreviations: MSC, mesenchymal stem/stromal cell; EV, extracellular vesicle; UC, umbilical cord; WJ, Wharton’s jelly; T1D/T2D, type 1/type 2 diabetes; DFU, diabetic foot ulcer; ED, erectile dysfunction; IV, intravenous; SOC, standard of care; CGM, continuous glucose monitoring; DLT, dose-limiting toxicity; AE/SAE, adverse/serious adverse event; MTD, maximum tolerated dose; RP2D, recommended phase 2 dose; IIEF-5, five-item International Index of Erectile Function; EHS, Erection Hardness Score. Registry product names do not independently establish EV biogenesis or marketing approval.
